# Site‐Specific Mitochondrial RNA N1‐Methyladenosine Demethylation via an Engineered MTS‐PUF‐ALKBH3 Fusion Protein

**DOI:** 10.1002/advs.202510482

**Published:** 2025-10-27

**Authors:** Xiangrui Li, Deqiang Kong, Hongmei Liu, Jie Yang, Jiale Zhou, Yuqiang Qian, Ding Zhao, Jinze Li, Xinyu Wu, Tao Zhang, Xiaodi Sun, Yang Han, Liangxue Lai, Zhanjun Li

**Affiliations:** ^1^ Laboratory of Organ Regeneration and Transplantation of The Ministry of Education, China‐Singapore Belt and Road Joint Laboratory on Liver Disease Research, State Key Laboratory for Diagnosis and Treatment of Severe Zoonotic Infectious Diseases The First Hospital of Jilin University Changchun 130062 China; ^2^ Jilin Provincial Key Laboratory of Animal Embryo Engineering, College of Animal Sciences Jilin University Changchun 130062 China; ^3^ Key Laboratory for Zoonosis Research of the Ministry of Education, Institute of Zoonosis, and College of Veterinary Medicine JIlin University Changchun 130062 China; ^4^ Key Laboratory of Zoonosis Research, Ministry of Education, College of Veterinary Medicine

**Keywords:** ALKBH3, demethylation editor, Mitochondrial RNA, N1‐methyladenosine, PUF

## Abstract

Mitochondrial RNA N1‐methyladenosine (m^1^A) is a prevalent and reversible epitranscriptomic modification. While the biological roles of cytosolic m^1^A have been increasingly understood, the causal relationship between site‐specific mitochondrial m^1^A and phenotypic outcomes remain elusive, partly due to the lack of precise editing tools. Here, a CRISPR‐free mitochondrial RNA m^1^A demethylation (MRD) editor is reported, which fuses mitochondria‐localized engineered PUF RNA‐binding protein with the m^1^A demethylase ALKBH3. Independent cellular assays across multiple sites confirm that MRD editor enables precise demethylation of m^1^A in mitochondrial mRNAs and tRNAs, leading to correlated changes in mitochondrial protein levels with minimal off‐target effects. The MRD editor is further employed to systematically investigate how site‐specific mitochondrial m^1^A alterations regulate cell proliferation, ATP production, mitochondrial membrane potential (MMP), and mitochondrial respiration. Finally, in vivo application of the MRD editor reveals that demethylation of m^1^A at the A9 position of mitochondrial tRNA‐Lys (MT‐TK9) induces severe immunodeficiency phenotypes in mice, as evidenced by transcriptomic and histopathological analyses. Collectively, the findings establish MRD as a versatile tool for site‐specific mitochondrial RNA m^1^A editing, offering new insights into the functional dissection of these modifications through chemical biology strategies.

## Graphical abstract

A CRISPR‐free mitochondrial RNA m^1^A demethylation (MRD) editor combining MTS, PUF proteins, and ALKBH3 enables precise m^1^A removal from mitochondrial mRNA and tRNA, which influences mitochondrial protein levels, cellular proliferation, ATP production, and mitochondrial respiration. Subsequently, in vivo demethylation of the m^1^A modification is achieved at mt‐tRNA‐Lys^A9^ using the MRD and obtained MRD‐TK9 and DIP‐MRD‐TK9 mice with immunodeficiency phenotypes.

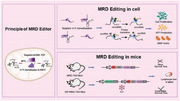

## 1 Introduction

N1‐methyladenosine (m^1^A) is a prevalent and dynamic RNA modification that plays a critical role in regulating diverse cellular processes and disease progression.^[^ ^1^ ^]^ In particular, studies have shown that m^1^A modification in mitochondrial ND5 mRNA can induce strong mitochondrial ribosome stalling, and m^1^A modifications in mt‐tRNA‐Lys^A9^ are required for the formation of the cloverleaf structure.^[^ ^2^, ^3^, ^4^ ^]^ Abnormal m^1^A modifications in mt‐RNAs have been implicated in diseases such as myoclonic epilepsy with ragged‐red fibers (MERRF), Alzheimer's disease, and multiple cancers.^[^ ^5^, ^6^, ^7^, ^8^, ^9^ ^]^ Despite an increasing recognition of the importance of mitochondrial m^1^A in cellular and individual fates, our understanding of its specific functions remains limited relative to cytoplasmic m^1^A, partly due to the absence of methods for editing site‐specific m^1^A modifications in mt‐RNAs.

RNA modification editing tools have been developed by engineering CRISPR/Cas systems fused with RNA methyltransferases or demethylases, enabling programmable post‐transcriptional editing of specific RNA sites.^[^ ^10^, ^11^, ^12^, ^13^, ^14^, ^15^, ^16^, ^17^, ^18^, ^19^, ^20^ ^]^ However, these approaches are severely limited in manipulating mitochondrial RNA modifications. The primary obstacle lies in the requirement for guide RNA (gRNA) assembly in CRISPR‐based systems, as gRNA struggles to penetrate the double membrane of mitochondria.^[^ ^21^, ^22^ ^]^ Thus, identifying CRISPR‐independent RNA‐binding proteins may offer a viable solution.

The Pumilio and FBF (PUF) homology protein family represent one such class of programmable RNA‐binding proteins that operate without gRNA assembly. A typical PUF domain consists of an N‐terminal region, a C‐terminal region, and eight structural repeats. Each repeat, consisting of 36 amino acids, recognizes a specific nucleotide through three key amino acid residues. This modular design allows the domain to be reprogrammed to target various 8‐nucleotide RNA sequences.^[^ ^23^, ^24^, ^25^, ^26^, ^27^, ^28^, ^29^ ^]^ PUF‐based fusion tools have been demonstrated to detect cellular RNAs, manipulate RNA transcription, splicing, translation, degradation, base editing, and m^6^A or m^7^G methylation or demethylation in vitro.^[^ ^29^, ^30^, ^31^, ^32^, ^33^, ^34^, ^35^ ^]^ Therefore, we speculated that tethering a customized PUF to the m^1^A “eraser” ALKBH3 and a mitochondrial targeting sequence (MTS) could enable site‐specific removal of m^1^A modifications within mt‐RNAs.

Here, we report a site‐specific mitochondrial RNA m^1^A demethylation editor, named MRD, created by fusing the RNA‐binding scaffold of the PUF protein to the m^1^A demethylase ALKBH3 and an MTS. MRD efficiently demethylated targeted mitochondrial mRNA and tRNA in cells and exhibited minimal off‐target editing activity. We further demonstrated its utility in assessing the functional consequences of mitochondrial m^1^A demethylation, including effects on cell proliferation and mitochondrial function. Moreover, we applied the MRD editor to successfully eliminate m^1^A modifications at the A9 site of mitochondrial tRNA‐Lys in mice and observed its significant effects on adaptive immunity development. By enabling programmable demethylation of mitochondrial RNA m^1^A both in vivo and in vitro, MRD is expected to better elucidate the biological and pathological implications of site‐specific mitochondrial RNA modifications.

## 2 Results

### 2.1 Demethylation of Mitochondrial mRNA by MRD

**Figure 1:**
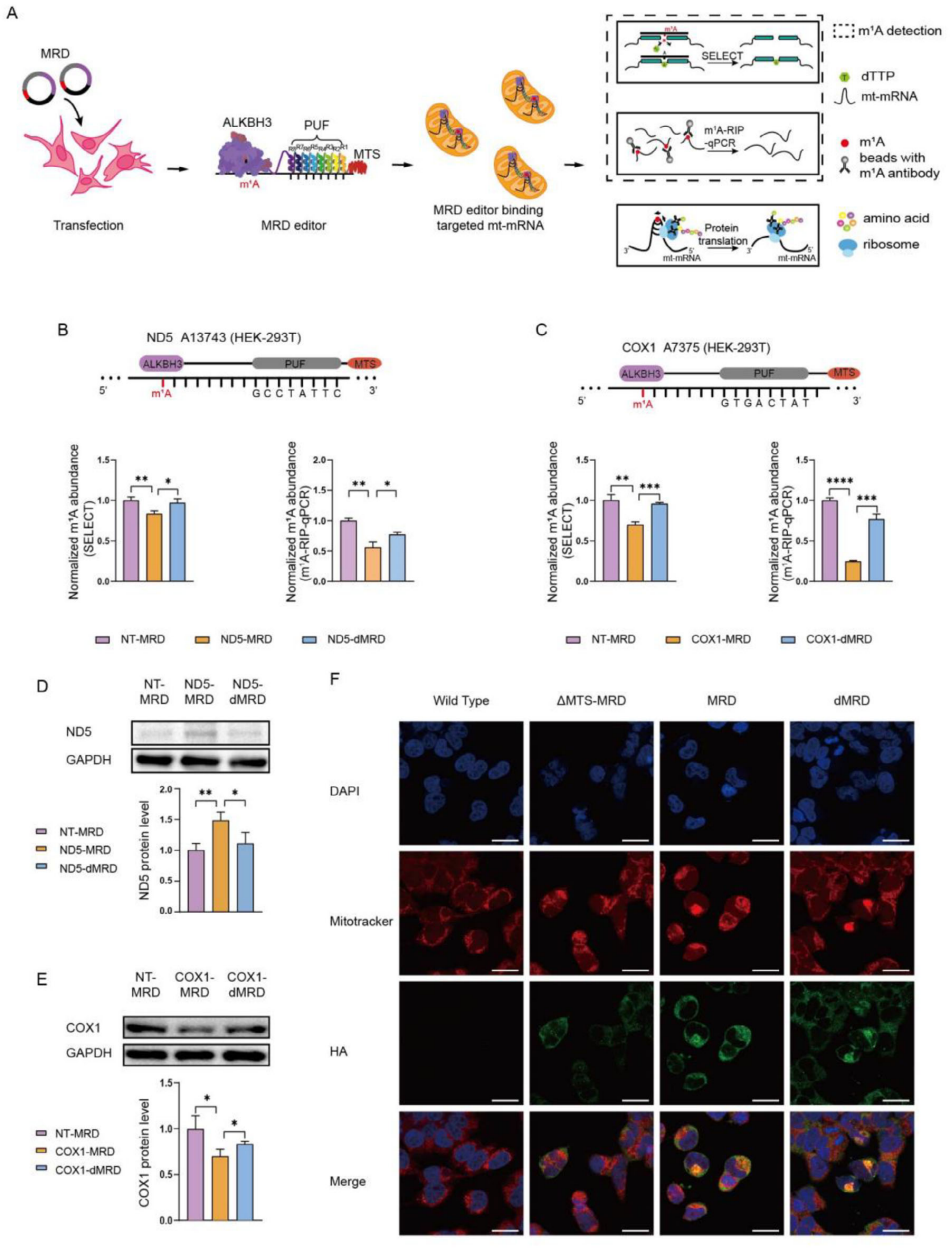
Plasmid construction and validation of MRD editor in mitochondrial mRNAs of HEK‐293T cells. A) Schematic representation of targeted mt‐mRNA demethylation by MRD, the identification of m^1^A methylation levels by RT‐1306、SELECT and m^1^A‐RIP‐qPCR, and the subsequent effects of m^1^A demethylation in protein translations. B,C) Normalized abundance of m^1^A at ND5 A13743 (B) and COX1 A7375 (C) by SELECT and m^1^A‐RIP‐qPCR in HEK‐293T cells. Statistical significances in SELECT and m^1^A‐RIP‐qPCR were calculated by unpaired two‐tailed Student's *t*‐test. (n = 3 biological replicates). D,E) Protein expression of ND5 (D) and COX1 (E) in HEK‐293T cells transfected with MRD plasmid targeted ND5 A13743 and control plasmid was checked by western blot analysis and quantitatively analyzed. Statistical significances in Western Blot analysis were calculated by unpaired two‐tailed Student's *t* ‐test. (n = 3 biological replicates). F) Immunofluorescence images of HEK‐293T cells transfected with MRD, deactivated MRD (dMRD) and MTS absent MRD (△MTS‐MRD), separately. Green = HA tag; blue = DAPI; red = Mito Tracker. Scale bar = 100 nm.

To develop a tool for studying site‐specific mitochondrial RNA m^1^A modification, we fused the PUF domain with MTS from subunit VIII‌ of mammalian cytochrome c oxidase, and the m^1^A eraser, ALKBH3, creating the MRD editor (**Figure** **1**A; Figure S1A,B, Supporting Information). First, to determine the subcellular location of the MRD editor, HEK‐293T cells were transduced and analyzed by confocal imaging. A high degree of colocalization between the red (mitochondria) and green (MRD) fluorescence confirmed its capacity to access mitochondrial transcripts (Figure 1F).

Then to evaluate the demethylation ability of MRD editor in mitochondrial mRNA, we selected the known m^1^A site at A13743 in mitochondrial ND5 mRNA.^[^ ^36^ ^]^ The PUF domain was engineered to target ND5 mRNA and MRD expressed in HEK‐293T cells. As controls, we generated PUF with demethylase‐dead ALKBH3 D193A (dALKBH3) and non‐targeting (NT) PUF with ALKBH3 to rule out the non‐specific effects of MRD editors. Based on previous report, we choose three methods to assess site‐specific m^1^A modification, including an evolved HIV reverse transcriptase RT‐1306 assay to induce A mutation at m^1^A sites during reverse transcription, an established single‐base elongation‐ and ligation‐based qPCR amplification method, termed SELECT, and m^1^A‐RNA immunoprecipitation qPCR (m^1^A‐RIP‐qPCR). We observed that HEK‐293T cells expressing MRD exhibited significant m^1^A reduction at ND5 A13743, as quantified by RT‐1306, SELECT, and m^1^A‐RIP‐qPCR (Figure 1B; Figure S2A, Supporting Information). Correspondingly, an upregulation of ND5 protein level was observed upon demethylation (Figure 1D), consistent with the conclusions that m^1^A in ND5 transcript can halt gene translation reported in previous studies.^[^ ^9^ ^]^

To explore the relationship between PUF binding position and editing efficiency, we generated multiple MRD variants targeting various distances around A13743 in ND5 mRNA. As shown in Figure S2B (Supporting Information), robust m^1^A editing was observed over a broad range spanning at least 20 nucleotides upstream and downstream of the PUF binding site, indicating that the MRD platform tolerates diverse binding–editing separations rather than requiring a narrowly defined spacing. Based on previous studies, we also programmed MRD to demethylate the low‐abundance m^1^A at A7375 of COX1 mRNA in HEK‐293T cells. Although RT‐1306 was insensitive at this site, SELECT and m^1^A‐RIP‐qPCR confirmed a significant decrease in m^1^A (Figure 1C) and a reduction in COX1 protein following MRD expression (Figure 1E).

To further evaluate whether MRD can demethylate mitochondrial mRNA m^1^A modifications across multiple cell lines, we applied MRD to ND5 and COX1 mRNAs in HepG2 cells. Compared to the control group, both targets exhibited comparable demethylation (Figures S2A and S3A,C, Supporting Information) and protein‐level changes (Figure S3B,D, Supporting Information). Together, these results across two target transcripts demonstrate that the MRD can site‐specifically remove mitochondrial mRNA m^1^A modifications and subsequently affect gene translation in various cell types.

### 2.2 Demethylation of Mitochondrial tRNA by MRD

**Figure 2:**
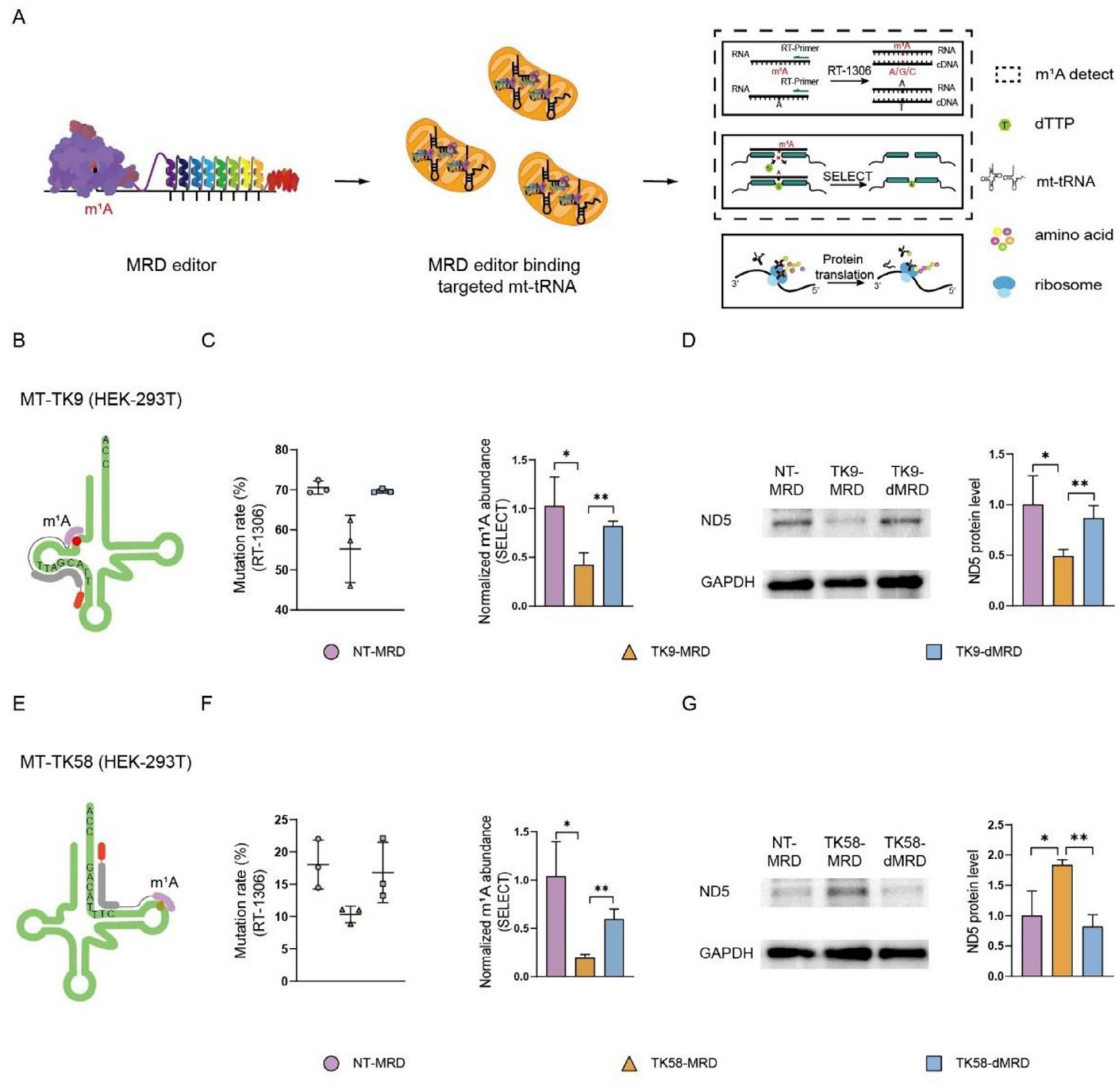
Validation of MRD editor in mitochondrial tRNAs of HEK‐293T cells. A) Schematic representation of targeted mt‐tRNA demethylation by MRD, the identification of m^1^A methylation levels by RT‐1306 and SELECT, and the subsequent effects of m^1^A demethylation in protein translations. B,E) Diagram of m^1^A localization and MRD targeting in MT‐TK9 (B) and MT‐TK58 (F). C,F) RT‐1306 (left panel) and SELECT (right panel) analysis of m^1^A in MT‐TK9 (C) and MT‐TK58 (F) site in HEK‐293T cells. D,G) Normalized abundance of m^1^A at MT‐TK9 (D) and MT‐TK58 (H) detected by SELECT in HEK‐293T cells. D,G) Protein expression of ND5 in HEK‐293T cells transfected with MRD plasmid targeted MT‐TK9 (D) or MT‐TK58 (G) and control plasmid was checked by western blot analysis and quantitatively analyzed.

The presence of m^1^A modifications in mitochondrial transfer RNAs (mt‐tRNAs) is essential for preserving structural integrity and functional competence.^[^ ^37^, ^38^, ^39^ ^]^ While PUF proteins are well‐established as canonical single‐stranded RNA (ssRNA)‐binding proteins, recent evidence indicates their capacity to bind RNA within substantially double‐stranded structures, albeit with reduced binding affinity.^[^ ^25^, ^40^ ^]^ Therefore, we sought to determine whether our MRD editors could effectively target mt‐tRNA and demethylate m^1^A (**Figure** **2**A). Previous studies have identified two high‐abundance m^1^A modification sites in mt‐tRNA‐Lys^A9^ (MT‐TK9) and A58 (MT‐TK58) (Figure 2B,E).^[^ ^39^, ^41^ ^]^ The MRD editors targeting MT‐TK9 and MT‐TK58 were first transfected into HEK‐293T cells. Notably, the methylation levels of the targeted transcripts decreased following transfection (Figure 2C,F).

Given the pivotal role of mt‐tRNA in protein synthesis, we investigated whether demethylation could alter protein levels. Each ND5 gene harbors 21 lysine codons, the highest number among mitochondrial protein‐coding genes. Additionally, ND5 protein has been reported to be implicated in multiple complex disorders, such as Leber's hereditary optic neuropathy, MELAS syndrome, Leigh syndrome, and various types of cancers.^[^ ^42^, ^43^, ^44^, ^45^, ^46^ ^]^ Therefore, we selected ND5 as a representative mitochondrial protein. Demethylation at the A9 site of mt‐tRNA‐Lys led to decreased ND5 protein levels (Figure 2D), while demethylation at the A58 site led to an increase in ND5 protein levels (Figure 2G). Consistent results were observed in HepG2 cells, where MRD editors similarly reduced m^1^A levels at both sites (Figure S4A,C, Supporting Information) and led to corresponding changes in ND5 protein levels (Figure S4B,D, Supporting Information). Collectively, these findings suggest that the MRD editor can effectively demethylate m^1^A at specific tRNA sites in mitochondria, thereby affecting protein synthesis.

### 2.3 Assessment of Off‐Target Effects of the MRD Editor

**Figure 3:**
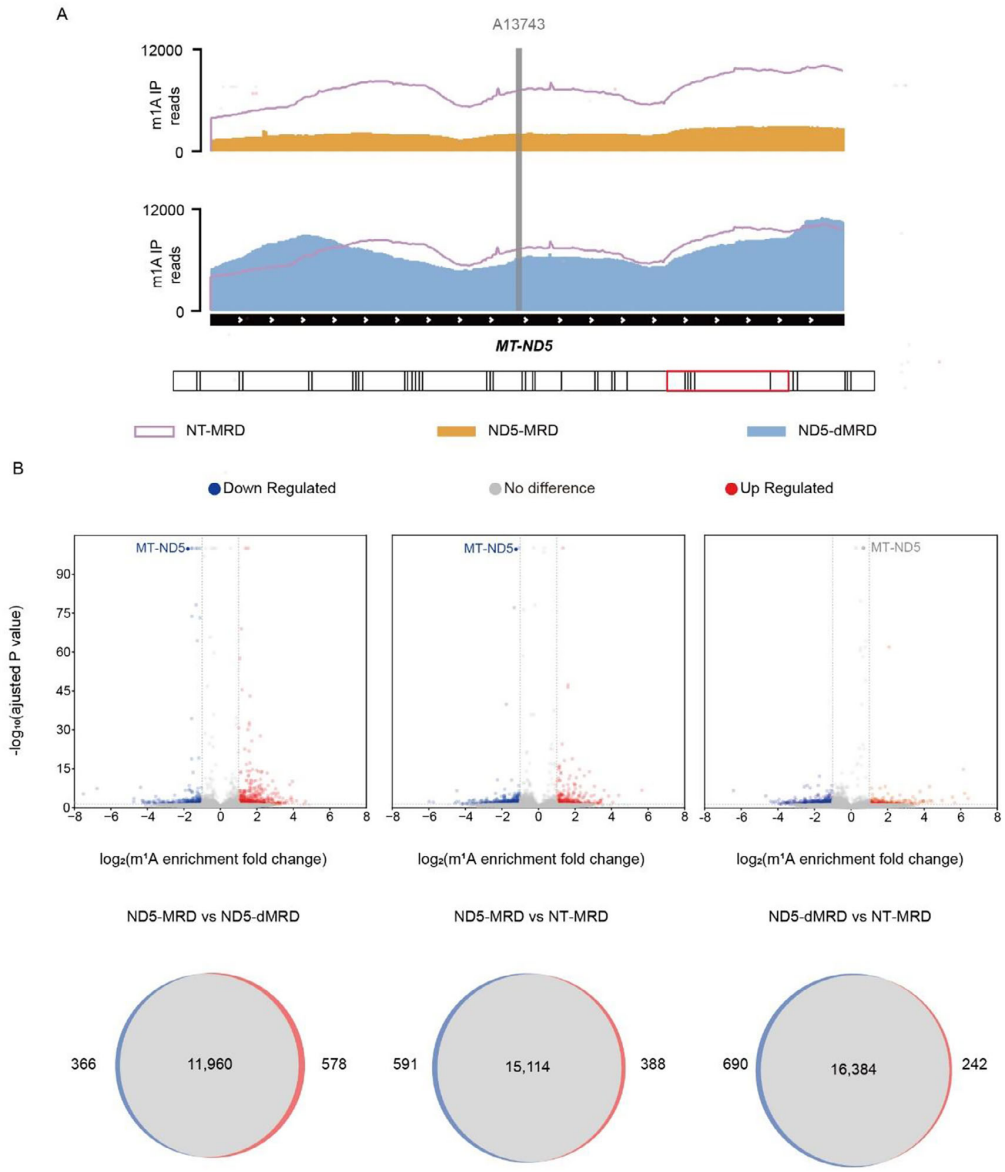
Assessment of Off‐Target Effects of the MRD Editor. A) Mitochondrial gene read coverage of m^1^A‐immunoprecipitated and input RNA from m^1^A‐RIP‐seq of transfected HEK‐293T cells. The ND5 A13743 site was specifically targeted using active MRD (ND5‐MRD), its catalytically inactive control (ND5‐dMRD), or non‐targeting control (NT‐MRD). B) Differential m^1^A enrichment of >12 900 methylated sites in HEK‐293T cells transfected with ND5‐MRD, ND5‐dMRD or NT‐MRD. Top panel: Volcano plots of differentially methylated m1A sites between ND5‐MRD versus ND5‐dMRD, ND5‐MRD versus NT‐MRD, and ND5‐dMRD versus NT‐MRD. Bottom panel: Venn diagrams illustrating the overlap of all methylated m1A sites among these groups.

To ensure that MRD activity does not result in substantial non‐specific demethylation, we next characterized its transcriptome‐wide off‐target editing. First, we evaluated the effect of MRD demethylation on the total m^1^A content within HEK‐293T cells via m^1^A‐RNA‐seq. Compared to a demethylase‐inactive control, MRD targeting ND5 A13743 significantly reduced m^1^A levels in the ND5 transcript, confirming efficient on‐target demethylation (**Figure** **3**A). Analysis of the remaining m^1^A sites showed that MRD induced ≈ 1000 m^1^A sites out of >12900 detected sites (∼7%), indicating minimal off‐target activity (Figure 3B).

To evaluate whether off‐target demethylation significantly altered the transcriptome, we conducted RNA‐seq‐based differential expression analysis between MRD‐transfected and control cells. Among the 26516 identified transcripts, 65 were down‐regulated and 78 were up‐regulated (padj. <0.05 and fold change >2) in the MRD group (Figure S5A, Supporting Information).

We further examined off‐target effects using MRD targeting the MT‐TK9 site. RNA‐seq analysis of transfected cells revealed 2 down regulated and 56 up regulated transcripts (out of total 16996) with pronounced alterations (padj. <0.05 and fold change >2) compared to the inactive control (Figure S5B, Supporting Information).

### 2.4 m^1^A Perturbation by MRD Alters Cell Growth and Mitochondrial Function

Mitochondrial gene expression exerts profound regulatory control over both mitochondrial functionality and cell fate determination.^[^ ^47^, ^48^ ^]^ Building on our previous findings that mitochondrial m^1^A hypomodification triggers dynamic changes in gene expression, we sought to investigate the capacity of the MRD editor to modulate cellular phenotype and mitochondrial function.

**Figure 4:**
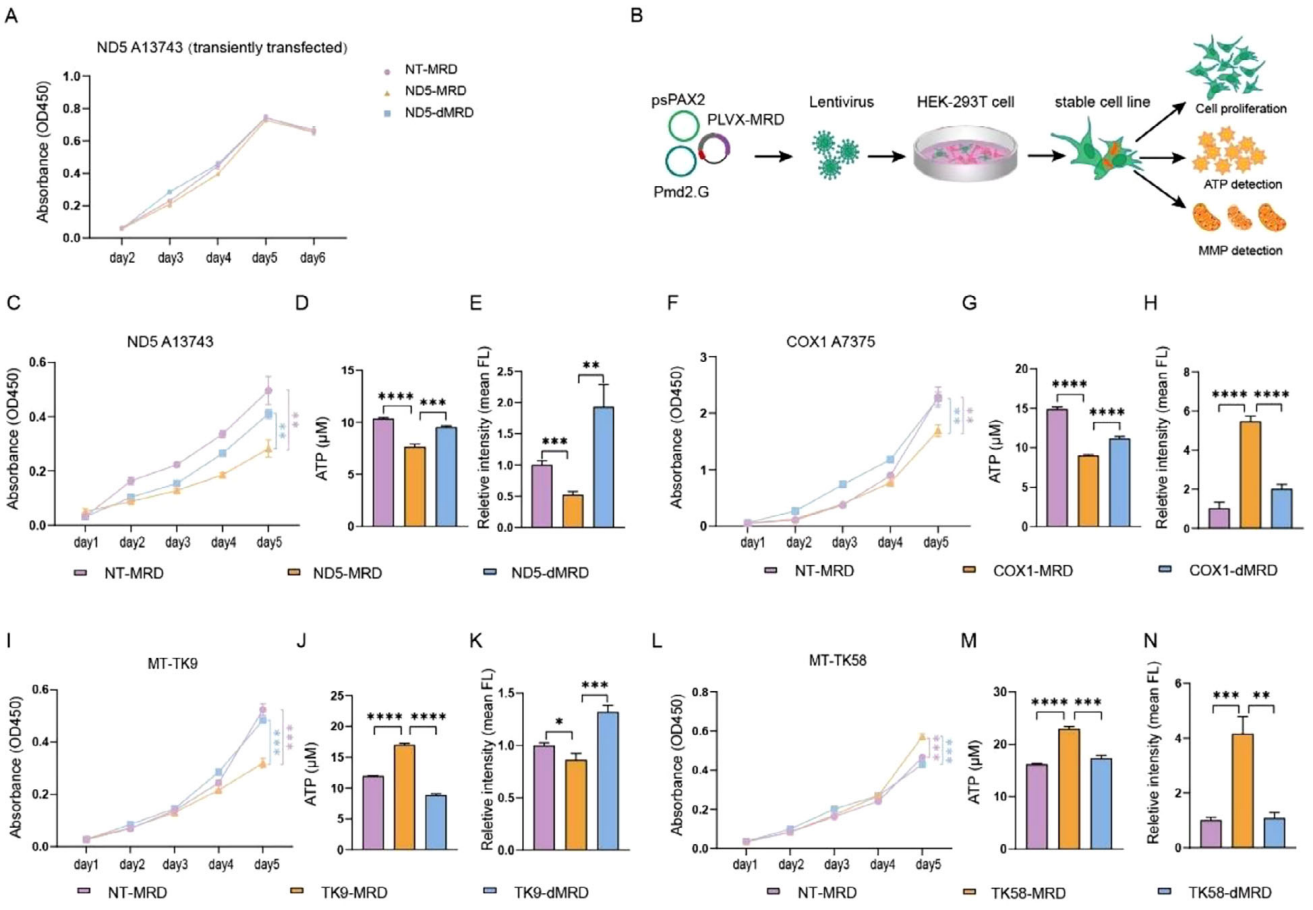
Alteration of cellular phenotypes by targeted m^1^A modification using the MRD editor. A) No significant alterations in cell proliferation following transient transfected with the ND5‐targeting MRD editor compared to the control group. B) Schematic representation of the delivery of MRD systems via lentiviral vectors to construct cell lines with consistent expression of the MRD editor. The effects on cellular phenotypes, including cell proliferation, ATP levels, and MMP, were separately assessed. C,F,I,L) Alterations in cell proliferation following targeted demethylation of ND5 A13743 (C), COX1 A7375 (F), MT‐TK9 (I) and MT‐TK58 (L) by the MRD editor, compared to the non‐targeted (NT) group and ALKBH3‐inactivated group in HEK‐293T cells. Statistical significance was determined by the unpaired *t*‐test (^*^ *p*< 0.05, ^**^ *p*< 0.01, ^***^ *p*< 0.001; ns, not significant). D,G,J,M) Alterations in ATP levels following targeted demethylation of ND5 A13743 (D), COX1 A7375 (G), MT‐TK9 (J) and MT‐TK58 (M) by the MRD editor, compared to the non‐targeted (NT) group and ALKBH3‐inactivated group in HEK‐293T cells. Statistical significance was determined by the unpaired *t*‐test (^*^ *p*< 0.05, ^**^ *p*< 0.01, ^***^ *p*< 0.001; ns, not significant). E,H,K,N) Alterations in MMP levels following targeted demethylation of ND5 A13743 (E), COX1 A7375 (H), MT‐TK9 (K) and MT‐TK58 (N) by the MRD editor, compared to the non‐targeted (NT) group and ALKBH3‐inactivated group in HEK‐293T cells. Statistical significance was determined by the unpaired *t*‐test (^*^ *p*< 0.05, ^**^ *p*< 0.01, ^***^ *p*< 0.001; ns, not significant).

In our initial assessment, HEK‐293T cells transiently transfected with the ND5‐targeting MRD editor showed no significant effect on proliferation over six days compared to controls (**Figure** **4**A). Since m^1^A is a dynamically regulated modification with rapid turnover kinetics, so we established stably transduced cell lines with genomically integrated MRD plasmid targeted to ND5 A13743, COX1 A7375, MT‐TK9, MT‐TK58, NT RNA and a version of inactive ALKBH3 with CoGFP sequence using a lentiviral delivery system (Figure 4B). Notably, m^1^A demethylation of ND5 A13743, COX1 A7375 and MT‐TK9 induced cell growth arrest (Figure 4C,F,I), while demethylation of MT‐TK58 promoted cell proliferation (Figure 4L).

Given the ability to generate ATP and maintenance of mitochondria membrane potential (MMP) are indispensable for cellular viability and homeostasis,^[^ ^49^, ^50^ ^]^ we assessed ATP levels and MMP in the edited cells. Reduced methylation at ND5 A13743 and COX1 A7375 led to a decline in ATP synthesis (Figure 4D,G), whereas demethylation at MT‐TK9 and MT‐TK58 enhanced ATP production (Figure 4J,M). To evaluate MMP, we employed the Mito‐Tracker Red CMXRos staining and JC‐1 flow cytometry in HEK‐293T cells. Loss of m^1^A at ND5 A13743 and MT‐TK9 reduced MMP levels (Figure 4E,K; Figures S6B,C and S7A,C, Supporting Information), while demethylation at COX1 A7375 and MT‐TK58 elevated MMP (Figure 4H,N; Figures S6A,D and S3B,D, Supporting Information).

We further analyzed mitochondrial respiration using the Seahorse XF Analyzer. Compared with controls, TK9‐MRD cells exhibited elevated basal and maximal oxygen consumption rates (OCR), indicative of enhanced mitochondrial respiratory capacity, together with reduced extracellular acidification rates (ECAR), reflecting decreased glycolytic reliance (Figure S8A, Supporting Information). In contrast, ND5‐MRD cells showed markedly diminished OCR and elevated ECAR, suggesting a metabolic shift toward glycolysis (Figure S8B, Supporting Information). Collectively, these results demonstrate that site‐specific m^1^A demethylation in mitochondrial RNAs exerts distinct effects on cell proliferation, ATP production, MMP maintenance, and mitochondrial respiration, underscoring the regulatory role of RNA modifications in cellular physiology and mitochondrial function.

### 2.5 Demethylating of m^1^A at the Mitochondrial tRNA‐Lys^A9^ Site Potentially Induces Severe Immunodeficiency in Mice

To explore the ability of MRD's targeted demethylation in vivo, we selected the A9 site of mt‐tRNA‐Lys, which exhibits the most abundant m^1^A modification in mice mt‐tRNA.^[^ ^7^ ^]^ In vitro studies have established that the m1A modification at position 9 is crucial for the maturation of human mitochondrial tRNA‐Lys^[^ ^4^, ^51^ ^]^; however, its in vivo functional significance remains poorly understood.

**Figure 5:**
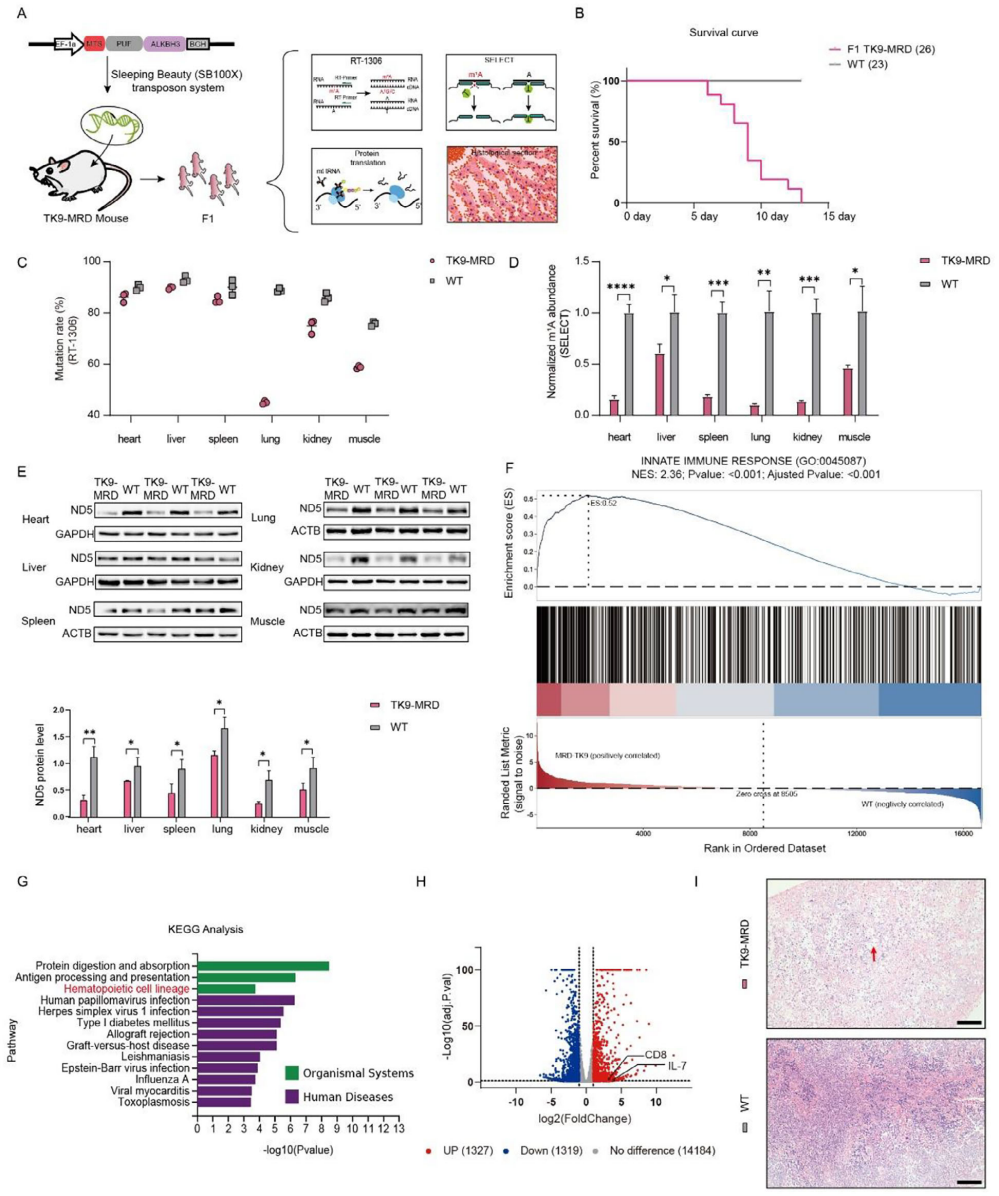
Demethylating m^1^A at mitochondrial tRNA‐Lys^A9^ site in mice. A) Schematic representation of the generation of the TK9‐MRD mice and the detection of m^1^A levels, protein levels and histological changes. B) Decreased survival rate of F1 generation TK9‐MRD mice compared to WT mice, shown by the survival curve. C,D) Decreased m^1^A levels in TK9‐MRD mice across multiple tissues detected by RT‐1306 (C) and SELECT (D). Statistical significance was determined using unpaired two‐tailed Student's *t*‐tests for all comparisons. (n = 3 mice). E) Protein expression of ND5 across multiple tissues was checked by western blot analysis and quantitatively analyzed. F) GO‐GSEA analysis of innate immune response pathway in the heart of TK9‐MRD and WT mice. G) Organismal systems and human diseases in terms of KEGG analysis in the heart of TK9‐MRD and WT mice. H) Volcano plots of differential expression genes in the heart of TK9‐MRD and WT mice. I) Histological changes in the spleen of TK9‐MRD mice compared to WT mice. Scale bar = 100 µm.

We employed the nonviral Sleeping Beauty (SB) 100X transposon system to deliver MRD plasmid targeted mouse mitochondrial tRNA‐Lys^A9^ m^1^A modification (mus‐TK9‐MRD plasmid) into the mouse genome.^[^ ^52^ ^]^ Initially, we cloned an expression cassette containing the mus‐TK9‐MRD plasmid, driven by the eukaryotic translation elongation factor 1 α (EF‐1α) promoter, into the SB transposon vector, creating the SB‐mus‐TK9‐MRD plasmid. The mus‐TK9‐MRD plasmid was then integrated into mouse zygotes by microinjection of SB‐X100 mRNA and SB‐mus‐TK9‐MRD plasmid. The injected embryos were then implanted into surrogate mothers, resulting in F0 offspring carrying the TK9‐MRD editor gene (TK9‐MRD mice) (**Figure** **5**A). PCR validation confirmed the presence of the TK9‐MRD gene in two out of five newborn (F0) mice (Figure S9A, Supporting Information). Unfortunately, one of these mice (F0‐1) died the day after birth, exhibiting symptoms such as small body size and cyanosis (Figure S9C, Supporting Information). Another mouse (F0‐2) grew to adulthood. We then mated the above adult mouse with two wild‐type (WT) mice and successfully obtained F1 TK9‐MRD mice (Figure S9A,B, Supporting Information).

Surprisingly, we observed that F1 TK9‐MRD mice progressively died within two weeks after birth (Figure 5B). To investigate the cause of the extremely low survival rate in TK9‐MRD mice, we humanely euthanized 1‐week‐old mice for subsequent analysis. First, to determine whether MRD can alter single site methylation in vivo, we assessed the m^1^A modification levels at the tRNA‐Lys A9 site in multiple organs by RT‐1306 methods and SELECT. Decreased methylation levels in TK9‐MRD mice were observed (Figure 5C,D, Supporting Information). Then, to determine if this demethylation at the mt‐tRNA‐Lys^A9^ site impaired mitochondrial protein ND5 synthesis in cells, we measured the levels of the mitochondrial ND5 protein in various organs. Western blot analysis demonstrated a significant downregulation of ND5 protein (Figure 5E). These findings confirm that the TK9‐MRD editor could effectively demethylate the targeted site in vivo and lead to impaired mitochondrial ND5 protein synthesis.

Subsequently, to further investigate the cause of the high mortality rate in TK9‐MRD mice, we performed RNA sequencing (RNA‐seq) on cardiac tissues from 1‐week‐old TK9‐MRD mice (n = 3) and their wild‐type littermates (n = 3). Combined GO and GSEA enrichment analysis revealed that the innate immune response‐associated genes were significantly enriched (P‐value < 0.001, normalized enrichment score = 2.36) in TK9‐MRD mice compared to wild‐type controls (Figure 5F), suggesting hyperactivation of innate immunity. Simultaneously, KEGG analysis revealed that disease pathways associated with various infections were enriched in TK9‐MRD mice (Figure 5G). Therefore, we hypothesize that the activation of the innate immune system in TK9‐MRD mice might be caused by infection. However, intriguingly, we found that T‐cell and B‐cell receptor signaling pathways were not enriched, indicating the absence of adaptive immune activation. This absence contrasts with the hyperactivated innate immune response, suggesting a dysregulated crosstalk between the two arms of immunity. Notably, our KEGG analysis revealed significant enrichment of the hematopoietic cell lineage pathway (Figure 5G), with multiple genes in the differentiation pathway of hematopoietic stem cells into various lymphocytes being upregulated (Figure 5H). The upregulation of genes such as IL‐7 and CD8 suggests the organism's sustained efforts to drive hematopoietic stem cell (HSC) differentiation into lymphocytes, implying a potential immunodeficiency in TK9‐MRD mice. This may represent one of the underlying causes that account for the significantly elevated mortality in TK9‐MRD mice compared to wild‐type mice.

To further validate this hypothesis, we conducted histopathological analysis on the spleen, the largest lymphoid organ in the body, via hematoxylin and eosin (HE) staining. We observed that the number of lymphocytes, hematopoietic and monocyte cells in white pulp was reduced, and the boundary between white pulp and red pulp was more unclear (Figure 5I). This finding further confirms immunodeficiency in TK9‐MRD mice. In summary, we demonstrate that the MRD editor achieves site‐specific m^1^A reduction at the A9 position of mitochondrial tRNA‐Lys in vivo. The resultant m^1^A loss likely drives increased mortality in mice by disrupting adaptive immune system development.

### 2.6 Doxycycline‐Induced Demethylation of m^1^A at the Mitochondrial tRNA‐Lys^A9^ Site in Mice

**Figure 6:**
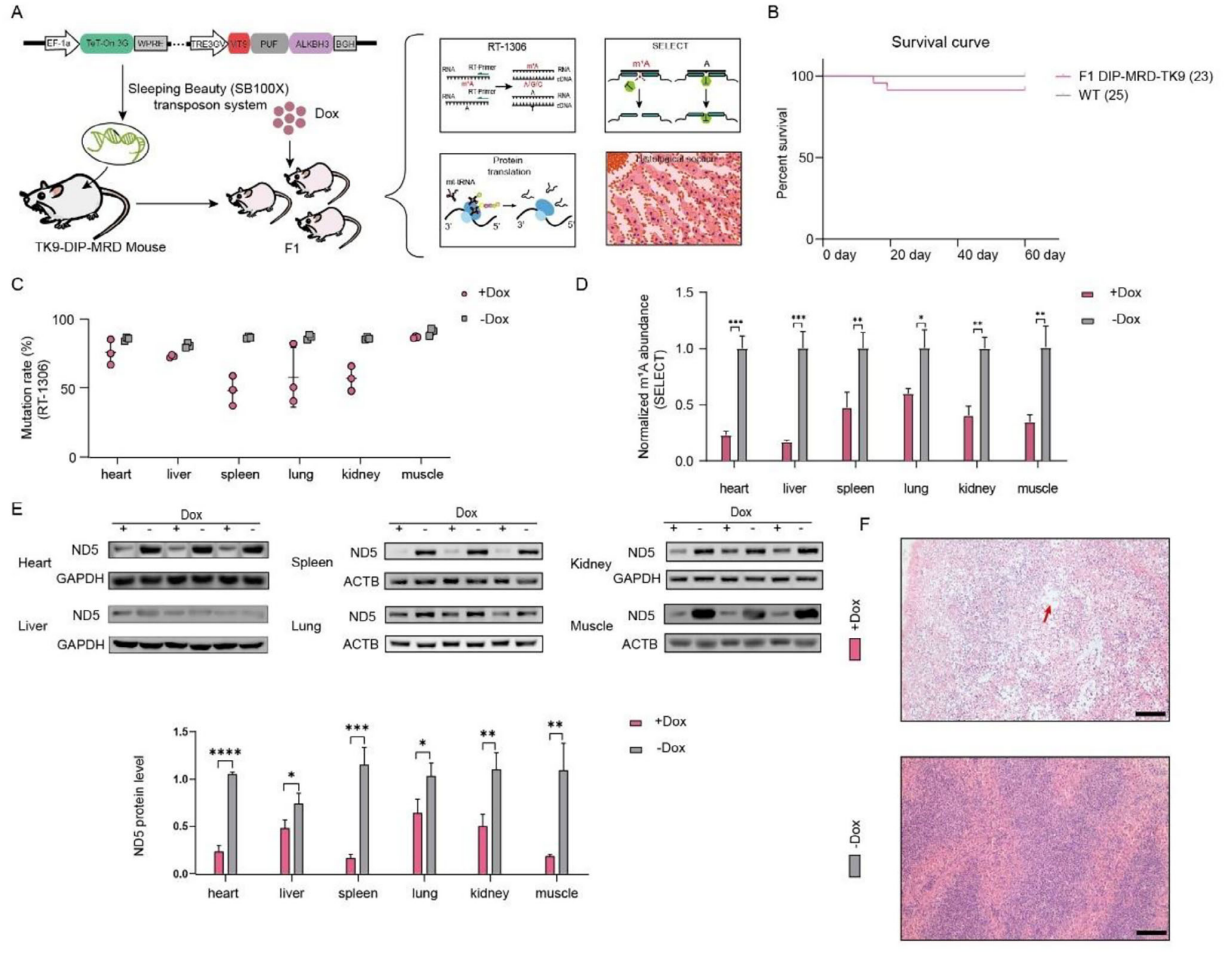
Dox‐induced demethylating m^1^A at mitochondrial tRNA‐Lys^A9^ site in mice. A) Schematic representation of the generation of TK9‐DIP‐MRD mice induced by Dox and the detection of m^1^A levels, protein levels and histological changes. B) Non‐significant difference in survival rate of F1 generation DIC‐TK9‐MRD mice compared to WT mice, shown by the survival curve. C,D) Decreased m^1^A levels in Dox‐induced TK9‐DIP‐MRD mice across multiple tissues detected by RT‐1306 (C) and SELECT (D). E) Protein expression of ND5 across multiple tissues was checked by western blot analysis and quantitatively analyzed. F) Histological changes in the spleen of Dox‐induced TK9‐DIP‐MRD mice compared to WT mice. Scale bar = 100 µm.

Next, to exclude the possibility that the high mortality in mice was caused by random integration of the SB transposon system, we utilized the Tet‐On system to generate a Dox‐inducible expression of the PUF‐based mus‐TK9‐MRD plasmid mouse model (TK9‐DIP‐MRD). We first constructed an rtTA and TRE3G‐controlled TK9‐MRD demethylation editor (**Figure** **6**A). Following the same methodology as the TK9‐MRD model, we obtained TK9‐DIP‐MRD mice in both F0 and F1 generations (Figure S10A,B, Supporting Information). Notably, untreated F1 TK9‐DIP‐MRD mice exhibited survival rates comparable to WT mice (Figure 6B), indicating that random transposon integration was not the primary cause of the high mortality in TK9‐MRD mice.

To assess the efficacy of m^1^A RNA demethylation, we administered Dox to 1‐month‐old TK9‐DIP‐MRD transgenic mice. Four weeks post‐treatment, we harvested multiple tissues for subsequent analysis. Results showed significant reductions in m^1^A levels at mt‐tRNA‐Lys A9 (Figure 6C,D) and mitochondrial ND5 protein expression (Figure 6E). Additionally, Dox‐induced TK9‐DIP‐MRD mice exhibited splenic pathologies similar to those in TK9‐MRD mice (Figure 6F), providing further evidence that immunodeficiency is primarily driven by MRD‐induced m^1^A hypomodification at mt‐tRNA‐Lys^A9^.

## 3 Discussion

Mitochondrial m^1^A is an essential RNA modification in mammals, influencing RNA metabolism and disease pathogenesis by regulating gene expression.^[^ ^6^, ^8^, ^9^ ^]^ While recent CRISPR‐based and methyltransferase tools have enabled site‐specific manipulation of m^1^A modifications in cytoplasmic transcripts,^[^ ^10^, ^11^ ^]^ editing mitochondrial m^1^A has remained challenging due to inefficient guide RNA import into mitochondria.^[^ ^21^, ^22^ ^]^ To address this key issue, we developed a CRISPR‐free mitochondrial RNA demethylation system by fusing programmable PUF domains with an m^1^A demethylase and mitochondrial targeting sequence.

This platform enables site‐specific m^1^A demethylation in mitochondrial mRNAs and tRNAs. It provides a targeted methodology to investigate the functional roles of single‐site modifications in diverse biological processes, eliminating the reliance on enzymatic knockout or overexpression.^[^ ^8^ ^]^ Notably, the MRD system features a modular architecture and can be easily adapted to target other mitochondrial RNA modifications by substituting the effector enzyme and the PUF recognition sequence. This system provides a versatile toolkit for elucidating the functional roles of site‐specific mitochondrial RNA modifications.

To validate the ability of the MRD editor in vivo and the function of m^1^A modification at A9 site in mt‐tRNA‐Lys, we generated TK9‐MRD and TK9‐DIP‐MRD mouse models. Both models exhibited severe splenic abnormalities, implicating that this site modification plays a critical role in the differentiation of hematopoietic stem cells (HSCs) toward lymphoid lineage cells. This aligns with emerging evidence that mitochondrial metabolism plays a key role in lineage differentiation^[^ ^53^ ^]^ though the underlying epitranscriptomic mechanisms remain poorly understood.

Furthermore, both TK9‐MRD and Dox‐induced TK9‐DIP‐MRD mouse models developed multi‐organ pathologies reminiscent of the features of mitochondrial dysfunction‐associated diseases (Figures S9D–I and S10C, Supporting Information),^[^ ^54^, ^55^, ^56^, ^57^, ^58^, ^59^, ^60^ ^]^ including hypertrophic cardiomyocytes, hepatocellular degeneration with lipid accumulation, pulmonary alveolar thickening and renal tubular epithelial injury. Notably, red ragged fibers (RRF), a hallmark of MERRF, were found in muscle tissue of TK9‐MRD mice (Figure S9H, Supporting Information). Neurological manifestations, including pallor of brain tissue (Figure S9I, Supporting Information), were also observed, consistent with mitochondrial encephalomyopathy, and closely resembling the pathological features of MELAS, MERRF, and Kearns‐Sayre syndrome.^[^ ^61^ ^]^ Besides, significant vacuole formation was noted in the muscle tissue of Dox‐induced TK9‐DIP‐MRD mice (Figure S10C, Supporting Information), which we hypothesize may be lipid droplets resulting from defects in fatty acid metabolism.^[^ ^62^ ^]^ These findings indicate a mechanistic link between dysregulated mitochondrial tRNA methylation and mitochondrial pathologies in vivo.

In light of its capacity to remove m^1^A marks from mitochondrial tRNA, MRD holds promise as a therapeutic tool for mitochondrial disorders, but its efficacy in disease models remains to be demonstrated. Future studies should identify pathogenic or disease‑modifying mitochondrial transcripts and then apply MRD‑TK9 to demethylate their m^1^A sites, thereby modulating gene expression for therapeutic benefit. Because our current editing efficiencies on mRNA substrates are relatively modest, further optimization of the MRD platform will be essential. For example, improving PUF‑mediated RNA binding affinity and discovering or engineering more active m^1^A demethylase domains may be required to achieve the activity levels necessary for in vivo applications.

In conclusion, our study presents MRD as a robust platform for programmable mitochondrial RNA demethylation. By enabling site‐specific editing of m^1^A in mitochondrial RNAs, MRD unlocks new avenues for mechanistic studies of mitochondrial epitranscriptomics. The MRD editor and accompanying mouse models will together pave the way for both basic research in mitochondrial biology and the development of novel therapeutic strategies for disorders.

## 4 Experimental Section

### Animals

ICR mice were obtained from the Laboratory Animal Center of Jilin University (Changchun, China). All animal studies were conducted according to experimental approaches and standards approved by the Animal Welfare and Research Ethics Committee at Jilin University. The Institutional Animal Care and Use Committee of Jilin University, China, provided ethical approval for this research (SY20231107).

### Plasmid Construction

The MRD editors were constructed by fusing COX8 MTS, eight repeats of PUF, and ALKBH3 to the backbone of PcDNA3.1. The eight repeats of PUF were chemically synthesized and assembled by PCR amplification. DNA fragments encoding the deaminase domains of the human ALKBH3 were amplified from the original “REMOVER” plasmid, which was gifted from Sichuan Normal University. To prevent interference between PUF and ALKBH3, a short linker was designed. Methyltransferase‐inactive editors were generated by introducing a D193A mutation in ALKBH3. The lentivirus‐associated plasmids were constructed by combining the PCR products of PUF with the backbone of a lentivirus plasmid.

Plasmid constructs used in this study were assembled using a 2 × Multif Seamless Assembly Mix (ABclonal) and Fast Site‐Directed Mutagenesis Kit (TIANGEN). Primers (Sangon Biotech) were employed to amplify DNA fragments using PrimeSTAR MAX DNA polymerase (Takara). The primers used in this study are listed in the Table S1 (Supporting Information) (Sangon Biotech, China).

### In Vitro Transcription

The plasmid pTS395_PhCMV‐SB100 (Addgene Plasmid #109053) was transcribed using the MAXIscript T7 Kit (Ambion) and purified with miRNeasy Mini Kit (Qiagen) according to the manufacturer's instructions. SB100 × mRNAs quality and concentration were measured by Nanodrop 2000 and agarose gel (1.5%) electrophoresis, respectively.

### Cell Culture and Transfection

HEK‐293T cells and HepG2 cells were cultured in Dulbecco's Modified Eagle Medium (Meilunbio) supplemented with 10% fetal bovine serum (CLARK) at 37 °C, 5% CO_2_.

Plasmid transfection was carried out using Hieff Trans Liposomal Transfection Reagent (YEASEN) following the manufacture's protocol. Cells were divided into six‐well plates equally and 8 h later, cells were transfected with 3 µg plasmid per well. Transfected cells were cultured under normal conditions.

### RT‐1306 Assay for m^1^A Detection

Total RNA (300 ng in 8 µL) was combined with 1 µL of 10 µm RT primer (see Table S2, Supporting Information), heated to 70 °C for 2 min, and then immediately placed on ice. The reverse transcription master mix was prepared by mixing 2 µL RT‑1306, 5 µL dNTP mix (2 mm each), and 4 µL of 5× RT buffer (250 mm Tris‑HCl, 1 m KCl, 10 mm MgCl_2_, 25 mm DTT), and added to the RNA–primer solution. Reverse transcription was carried out at 37 °C for 1 h, followed by an 80 °C incubation for 10 min to inactivate the enzyme.

The extended cDNA products were amplified by PCR (primers listed in Table S2, Supporting Information), then subjected to a second, dual‐index barcoding PCR in 96‑well format. Barcoded amplicons were quantified, normalized to equimolar concentrations, pooled, and sequenced on Illumina HiSeq platform.

The raw FASTQ data were analyzed via the Hi‐TOM web server () to directly retrieve mutation profiles. Following established protocol^[^ ^63^ ^]^ m^1^A levels were quantified as the A‑to‑T mutation rate, calculated as:

(1) $$m^{1}A\left(\%\right)=\frac{\mathrm{Number}\;\mathrm{of}\;T\;\mathrm{reads}\;\mathrm{at}\;\mathrm{site}}{\mathrm{Total}\;\mathrm{qualified}\;\mathrm{reads}\;\mathrm{at}\;\mathrm{site}}\times 100$$

### SELECT Assay for m^1^A Detection

Detection of m^1^A at targeted sites by SELECT was modified from a previous protocol.^[^ ^64^ ^]^ For each sample, 1 µg of total RNA was incubated with 40 nm Up primer, 40 nm Down primer, and 5 µm dNTP (Beyotime) in 17.5 µl 1 × Cutsmart buffer (NEB). A progressive annealing cycle was carried out: 1 min each at 90, 80, 70, 60, 50, and 40 °C for 6 mins. Subsequently, 2.5 µl of enzyme mixture containing 0.01 U Bst 2.0 DNA polymerase (NEB), 0.5 U SplintR ligase (NEB), and 10 nm ATP (NEB) were added to the 17.5 µL annealing products. The final 20 µL reaction mixtures were incubated at 40 °C for 20 min, denatured at 80 °C for 20 min and then kept at 4 °C. 2 µL of final products were then transferred to a reaction mixture containing 200 nm SELECT common primers and 2 × SYBR Green Master Mix (TIANGEN) for qPCR analysis. The run cycle was set up as: 95 °C for 1 min followed by 40 cycles of (95 °C, 20 s; 60 °C, 60 s). The SELECT products of targeted sites were normalized to the near non‐targeted sites. All assays were performed with three independent experiments. Primer sequences are listed in the Table S3 (Supporting Information).

### m^1^A‐RNA Immunoprecipitation qPCR (m^1^A‐RIP‐qPCR)

Total RNA was extracted from cells using TRIzol reagent, followed by DNase I treatment to eliminate DNA contamination. 40 µg of total RNA was incubated with 1 µg of anti‐ m^1^A antibody (Abcam, ab208196) in RIP buffer (150 mm NaCl, 0.1% NP‐40, 10 mm Tris, pH 7.4) at 4 °C overnight. The mixture was then incubated with 40 µl of Protein A/G beads, rinsed with RIP buffer, for an additional 4 h at 4 °C. Beads were washed five times with RIP buffer, and the precipitated RNA was purified using TRIzol as per the manufacturer's instructions (Invitrogen, USA). Input and immunoprecipitated RNAs were reverse transcribed into cDNA and quantified by RT‐qPCR. Primer sequences are listed in Table S5 (Supporting Information).

### m^1^A‐RIP‐seq

Total RNA was isolated from cells transfected with the MRD plasmid and subjected to quality assessment. The RNA was treated with DNase I at 37 °C for 10 min to remove residual DNA, followed by column‐based purification. The purified RNA was fragmented using RNA Fragmentation Reagents at 70 °C for 5 min, then column‐purified again. A fraction (1/20) of the purified RNA was retained as Input (IP:Input ratio = 20:1), while the remainder was used for immunoprecipitation (IP). 4 µg of m^1^A antibody (Synaptic Systems) was incubated with Dynabeads Protein A/G to form antibody‐bead complexes. The fragmented RNA was then incubated with the antibody‐bound beads to enrich m^1^A‐modified RNA fragments. The IP reaction mixture was washed thoroughly, and the RNA fragments were eluted and purified. Both the IP‐enriched RNA and 10 ng of Input RNA were processed using the SMARTer Stranded Total RNA‐Seq Kit v2 – Pico Input Mammalian for reverse transcription and library construction. The libraries were amplified via PCR, and size selection was performed using magnetic beads to obtain final sequencing libraries. The libraries were initially quantified using Qubit 2.0 and diluted to 1 ng µL^−1^. The insert size distribution was verified using an Agilent 2100 Bioanalyzer. Libraries meeting size criteria were further quantified via qPCR to ensure effective concentrations > 2 nm. Qualified libraries were pooled based on effective concentrations and sequencing depth requirements, followed by sequencing on the Illumina Nova platform with a paired‐end 150 (PE150) strategy.

Low‐quality bases were trimmed using Trimmomatic (v0.38). Clean reads were aligned to the human reference genome (hg38) using HISAT2 with default parameters. The resulting SAM files were converted to BAM format and subjected to the following filtering steps: 1) retaining only uniquely and properly mapped reads; 2) removing reads with low mapping quality (MAPQ < 30); and 3) excluding reads mapped to blacklist regions. Peak calling and differential peak analysis were performed using the R package exomePeak. Peaks with a *p*‐value < 0.05 and fold change > 1.5 were considered significantly differential.

### RNA‐seq

Polyadenylated mRNA was isolated from total RNA using Oligo(dT) magnetic beads to enrich RNA molecules with polyA tails. The enriched mRNA was fragmented into ≈300 bp fragments via ion‐mediated RNA fragmentation. Fragmented RNA was reverse‐transcribed into first‐strand cDNA using random hexamer primers and reverse transcriptase. Second‐strand cDNA was synthesized using the first‐strand cDNA as a template. The double‐stranded cDNA was amplified by PCR to enrich the library. Library fragments were size‐selected to ≈450 bp using magnetic bead‐based purification. Library integrity and size distribution were assessed using an Agilent Bioanalyzer. Total library concentration and effective molarity (molarity of amplifiable fragments) were quantified. Libraries with distinct index sequences were pooled proportionally based on their effective concentrations and desired sequencing depth. The pooled libraries were diluted to 2 nm and denatured into single‐stranded DNA using alkaline treatment. Denatured libraries were sequenced on the Illumina platform using a paired‐end (PE) 150 bp strategy as part of a Next‐Generation Sequencing (NGS) workflow.

Gene expression was quantified using StringTie with default parameters. Expression levels were calculated based on read counts, and normalized as TPM (Transcripts Per Million mapped reads) to estimate transcript abundance. Differentially expressed genes (DEGs) were identified using DESeq2. Genes with a *p*‐value < 0.05 and fold change > 1.5 were considered significantly differentially expressed.

### Lentivirus Packaging

In six‐well plates, HEK‐293T cells were transfected with 1 µg of psPAX2 (Addgene plasmid, #12260), 1 µg of Pmd2.G plasmid (Addgene plasmid, #12259), and 1 µg of proposed plasmid per well with Hieff Trans Liposomal Transfection Reagent. The transfected cells were incubated for 72 h, after which the cultured supernatant was collected and filtered. This filtered supernatant was then used to culture new HEK‐293T cells. After 48 h, the supernatant was replaced with normal culture medium containing puromycin at the appropriate concentration. After 72 h, the medium was replaced again with normal medium for continued cell culture.

### Immunofluorescence Microscopy

An HA tag was cloned onto the editor to validate the translocation of the editor into the mitochondria. The HA‐tagged plasmid was transfected into HEK‐293T cells in 24‐well plates. After 36 h, the medium was replaced with fresh medium containing Mito Tracker Red CMXRos (Beyotime), and the cells were incubated for 30 mins. The cells were then gently washed by PBS and fixed by 4% paraformaldehyde for 20 min, followed by three PBS washes. The cells were permeabilized with 0.2% Triton‐X100 for 20 min and washed three times with PBS. Blocking was performed with 10% fetal bovine serum for 2 h at room temperature. Cells were then incubated overnight at 4 °C with an Anti‐HA Tag rabbit polyclonal antibody (Sangon Biotech). The following day, the cells were washed three times by PBS and incubated with DyLight 488 Conjugated AffiniPure Goat Anti‐rabbit IgG (H+L) (BOSTER) for 1 h at room temperature. After three more PBS washes, the cells were stained with DAPI. Imaging was conducted using an Olympus FV3000 confocal microscopy, with excitation wavelengths of 405, 488, and 561 nm. A 60 × oil immersion lens was used for imaging and the images were analyzed by image J.

### Cell Proliferation Assay

Cell proliferation was assessed using Cell Counting Kit‐8 (Meilunbio). A total of 3000 cells were seeded into each well of a 96‐well plate and cultured in 100 µl of standard medium. Prior to measurement, the medium was replaced with 90 µl of fresh medium mixed with 10 µl CCK‐8 reagent. The cells were then incubated at 37 °C for 2 h. Absorbance at 450 nm was detected using an 800TS microplate reader (Biotek) to determine cell proliferation.

### Western Blot

Cells were lysed with RIPA Lysis Buffer supplemented with protease inhibitor cocktail (Roche, Basel, Switzerland) on ice for 30 mins and centrifuged 13 000 rpm min^−1^ for 15 min. Protein concentration was determined using BCA kit (Meilunbio), and samples were denatured at 100 °C in loading buffer. Proteins were then separated via sodium dodecyl sulphate‐polyacrylamide gel electrophoresis (SDS‐PAGE) and transferred onto a 0.45 µm polyvinylidene difluoride (PVDF) membrane. The following primary antibodies were used in this study: anti‐HA (Sangon Biotech, D110004), anti‐GAPDH Polyclonal (Proteintech, 10494‐1‐AP), anti‐ALKBH3 (Proteintech, 12292‐1‐AP), anti‐ACTB (Sangon Biotech, D610001), anti‐ND5 (Sangon Biotech, D162970), anti‐ND6 (Sangon Biotech, D261927), anti‐COX1(Bioss, bs‐3953R), and anti‐COX2(Bioss, bs‐10431R). Immunoreactive bands were visualized and captured using the Tanon 5200 Imaging System (Tanon).

### RNA Isolation and RT‐qPCR

RNA was extracted using TRIZOL reagent (TIANGEN) following the manufacturer's protocol. For each sample, 2 µg RNA was reverse‐transcribed into cDNA using FastKing gDNA Dispelling RT SuperMix (TIANGEN). RT‐qPCR was performed on QuantStudio3 (Thermo Fisher Scientific) using SuperReal PreMix Plus (SYBR Green) (TIANGEN). Each 20 µl qPCR reaction contained 1 µL of cDNA templates, 1 µm of Up and down primers and 10 µL of 2 × qPCR mastermix. All reactions were carried out in technical triplicate.

### Microinjection of Mouse Zygotes and Genotyping

Briefly, a mixture of SB100x mRNA (20 ng µL^−1^) and mus‐MT‐TK9 MRD plasmid (60 ng µL^−1^) was co‐injected into the cytoplasm of pronuclear‐stage zygotes. The injected zygotes were transferred to potassium simplex optimized medium (KSOM) for culture at 37 °C with 5% CO2 until the two‐cell stage. Then, ≈30 to 50 injected embryos were transferred into the oviduct of the recipient mother.

Genomic DNA was extracted in One Step Mouse Genotyping Kit (Vazyme PD101) at 55 °C for 30 mins and then at 95 °C for 5 mins in a Bio‐Rad PCR Amplifier. Then, the extracted products were amplified by PCR [95 C, 5 mins for pre‐degeneration; 38 cycles of (95 °C, 30 s; 58 °C, 30 s; 72 °C, 30 s); 72 °C, 5 mins for extension] and identified by Sanger sequencing. All primers used for genotyping are listed in Table S4 (Supporting Information).

### Pathological Studies

All kinds of tissues were fixed in 4% paraformaldehyde for 48 h, embedded in paraffin wax, and then sectioned to produce slides. The slides were stained with H&E and viewed under a Nikon ts100 microscope. Fresh skeletal muscle biopsy of the mice was used for modified Gomori trichrome (MGT) staining.

### MMP Measurement

Mitochondrial membrane potential (MMP) was assessed using two complementary approaches. For confocal microscopy, living cells were stained with MitoTracker Red CMXRos (Beyotime, Shanghai, China) at a final concentration of 200 nm for 30 min at 37 °C according to the manufacturer's instructions. Images were acquired using an Olympus FV3000 confocal microscope, and fluorescence intensity was quantified with ImageJ software.

For flow cytometry, cells were harvested, washed twice with PBS, and resuspended in culture medium at a density of 1 × 10^^^6 cells mL^−1^. JC‐1 dye (Beyotime) was added, and cells were incubated for 20 min at 37 °C in the dark. After two washes with PBS, samples were analyzed immediately on a BD FACSymphony A5 Cell Analyzer (BD Biosciences). JC‐1 monomers (green fluorescence) were detected at 530 nm and J‐aggregates (red fluorescence) at 590 nm. The red/green fluorescence ratio was calculated as an indicator of MMP. For each sample, at least 10000 events were collected. Data are presented as mean ± s.d. from three independent experiments.

### Measurement of Mitochondrial Activities

Mitochondrial activities were measured using an ATP assay kit (Beyotime) according to the manufacturer's instructions. After centrifugation to remove cell debris, the supernatant was added to the substrate solution. The luminescence was recorded using an illuminometer, with an integration time of 10 s per well.

### OCR and ECAR Measurements by Seahorse XF Analyzer

Cells were seeded at a density of 5 × 10^^^4 cells per well in XF24 plates and cultured overnight. Prior to the assay, cells were washed and incubated in XF base medium supplemented with 10 mm glucose, 2 mm glutamine, and 1 mm pyruvate (pH 7.4) at 37 °C without CO2 for 1 h. Oxygen consumption rate (OCR) and extracellular acidification rate (ECAR) were determined using the Seahorse XF24 analyzer. Mitochondrial respiration and glycolytic parameters were evaluated following the sequential addition of glucose (10 mm), oligomycin (1 µm), and 2‐deoxyglucose (2‐DG, 50 mm). Data are presented as mean ± s.d. from four independent experiments.

### Dox Treatment

Dox was administered to mice via drinking water at a concentration of 0.5 mg mL^−1^, mixed with 2 mg mL^−1^ of sucrose, for one month. Additionally, three intraperitoneal injections of Dox were given at a dose of 2 mg kg^−1^ over the course of 1 week.

### Statistical Analysis

Quantitative and statistical analyses were performed using GraphPad Prism 8 software. Each experiment was independently repeated at least three times, and data are presented as mean ± standard deviation (SD). Relative protein levels were normalized to the NT group, which was set as a reference value of 1 for all comparisons. Densitometric analysis was used for quantification of immunoblots. All experiments were randomized and analyzed in a blinded manner, with no specific inclusion or exclusion criteria applied.

For statistical comparisons, unpaired two‐tailed Student's *t*‐test was used when two groups were compared. For experiments involving more than two groups, one‐way analysis of variance (ANOVA) followed by Tukey's post hoc test was applied. A P value < 0.05 was considered statistically significant.

## Conflict of Interest

The authors declare no conflict of interest.

## Supporting information



Supporting Information

## Data Availability

The data that support the findings of this study are available in the supplementary material of this article.
