# Supplementary material for: Site‐Specific Mitochondrial RNA N1‐Methyladenosine Demethylation via an Engineered MTS‐PUF‐ALKBH3 Fusion Protein
Source: Adv Sci (Weinh). 2025 Oct 27;13(1):e10482. doi: 10.1002/advs.202510482 (PMC12767104; doi:10.1002/advs.202510482)
Supplement: Supplementary file 1 — Supporting Information [file ADVS-13-e10482-s001.docx]

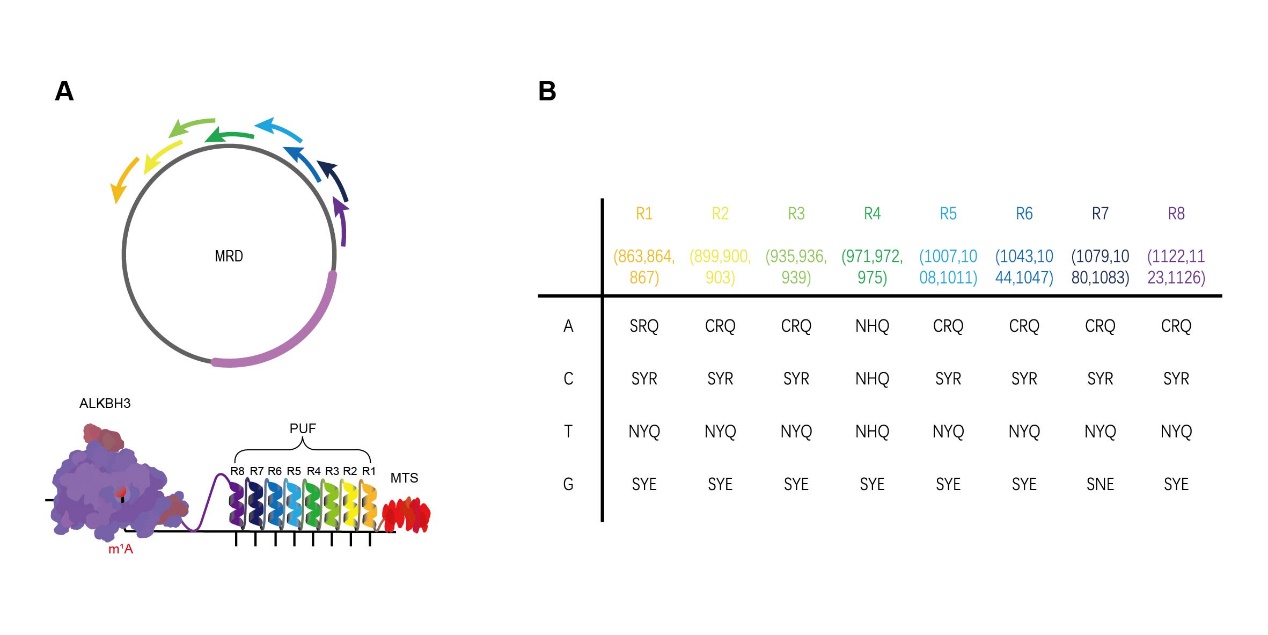


**Supplementary Figure S1.** **Design principles for the PUF targeting domain in MRD.**

(A) The MRD plasmid is composed of a mitochondrial targeting signal (MTS), the RNA-binding protein PUF, and the m¹A demethylase ALKBH3.

(B) A combinatorial library of key amino acids within each PUF repeat was designed to target specific nucleotides, allowing programmable recognition of different RNA sequences.

**
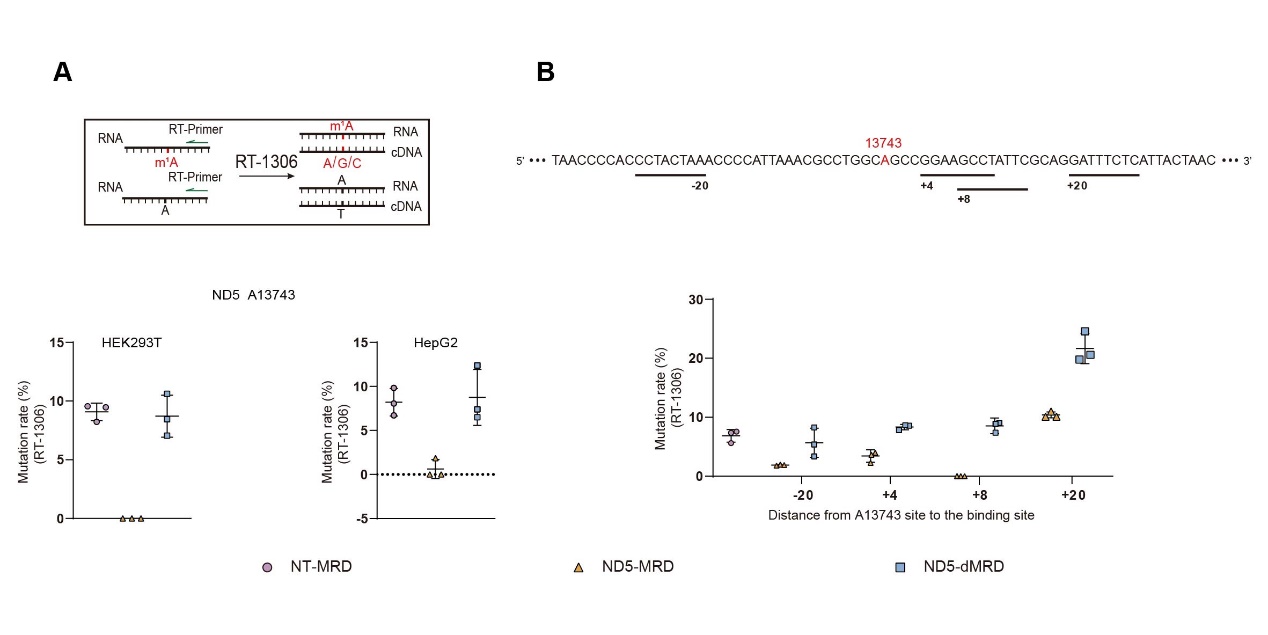
**

**Supplementary Figure S2. ND5 m^1^A modification abundance and MRD editing range analyzed via RT1306-PCR.**

(A) Mutation rate of m^1^A at site A13743 in ND5 detected by RT-1306 in HEK293T cells expressing non-targeting MRD (NT-MRD, purple circle), ND5-targeting MRD (ND5-MRD, yellow triangle) or deactivated MRD (ND5-dMRD, blue square)

(B) Editing efficiency of ND5 A13743 m^1^A modification by MRD binding at varying distances from the editing site. The mutation rate at A13743 is plotted against the distance from the editing site to the 5′ end of the PUF binding site for both MRD (ND5-MRD) and deactivated MRD (ND5-dMRD), compared with NT-MRD. Error bars represent ±SD from three independent replicates.

**
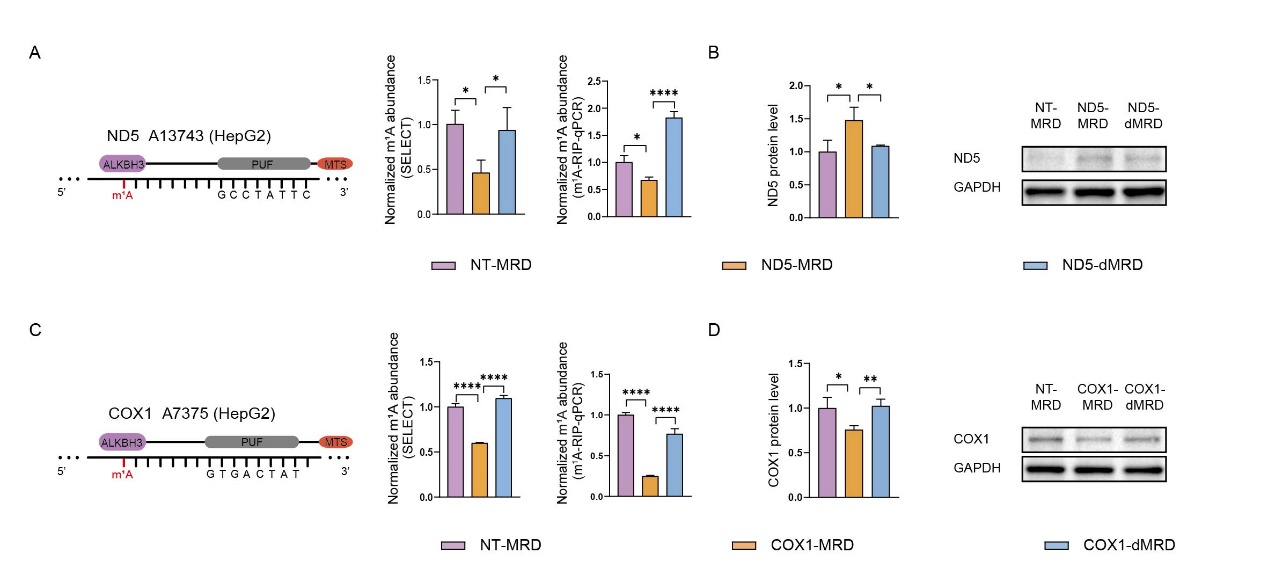
Supplementary Figure S3. Plasmid construction and validation of MRD editor in mitochondrial mRNAs of HepG2 cells.**

(A) Normalized abundance of m^1^A at ND5 A13743 by SELECT and m^1^A-RIP-qPCR in HepG2 cells.

(B) Protein expression of ND5 in HepG2 cells transfected with MRD plasmid targeted ND5 A13743 and control plasmid was checked by western blot analysis and quantitatively analyzed.

(C) Normalized abundance of m^1^A at COX1 A7375 by SELECT and m^1^A-RIP-qPCR in HepG2 cells.

(D) Protein expression of COX1 in HepG2 cells transfected with MRD plasmid targeting COX1 A7375 or control plasmid, examined by western blot and quantitatively analyzed.

**
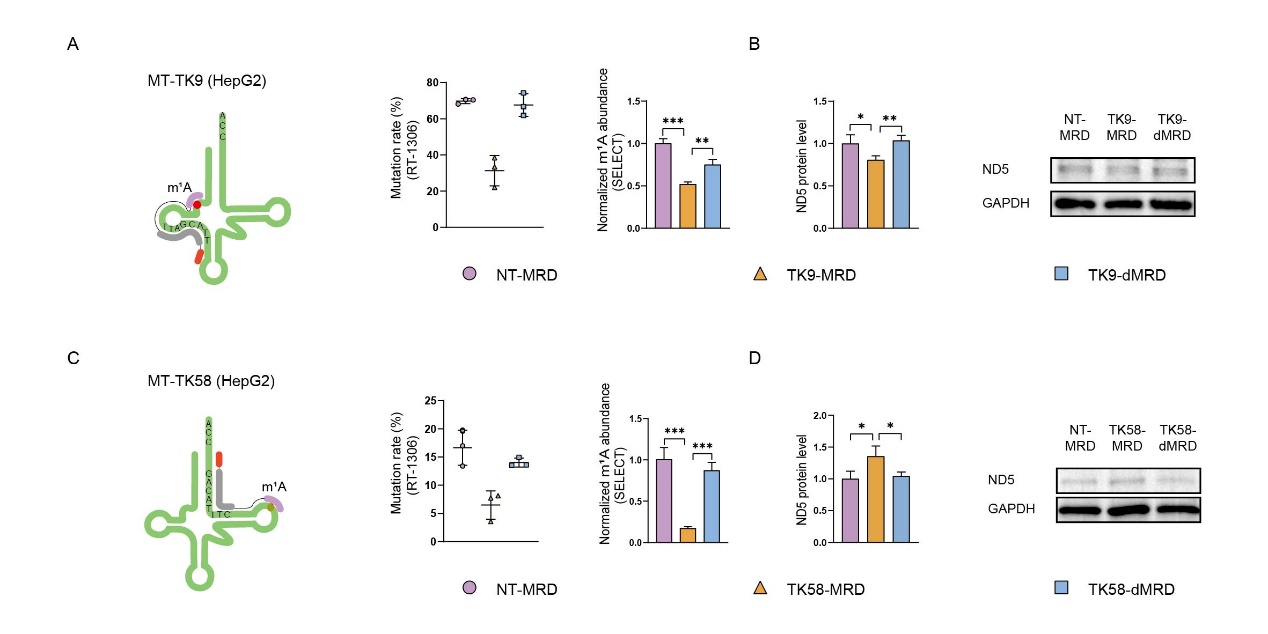
Supplementary Figure S4.** **Validation of MRD editor in mitochondrial tRNAs of HepG2 cells.**

(A) Mutation rate of m^1^A at MT-TK9 by RT-1306 in HepG2 cells. Normalized abundance of m^1^A at MT-TK9 by SELECT in HepG2 cells.

(B) Protein expression of ND5 in HepG2 cells transfected with MRD plasmid targeted mt-tRNA-Lys A9 and control plasmid was checked by western blot analysis and quantitatively analyzed.

(C) Mutation rate of m^1^A at MT-TK58 by RT-1306 in HepG2 cells. Normalized abundance of m^1^A at MT-TK58 by SELECT in HepG2 cells.

(D) Protein expression of ND5 in HepG2 cells transfected with MRD plasmid targeted MT-TK58 and control plasmid was checked by western blot analysis and quantitatively analyzed.


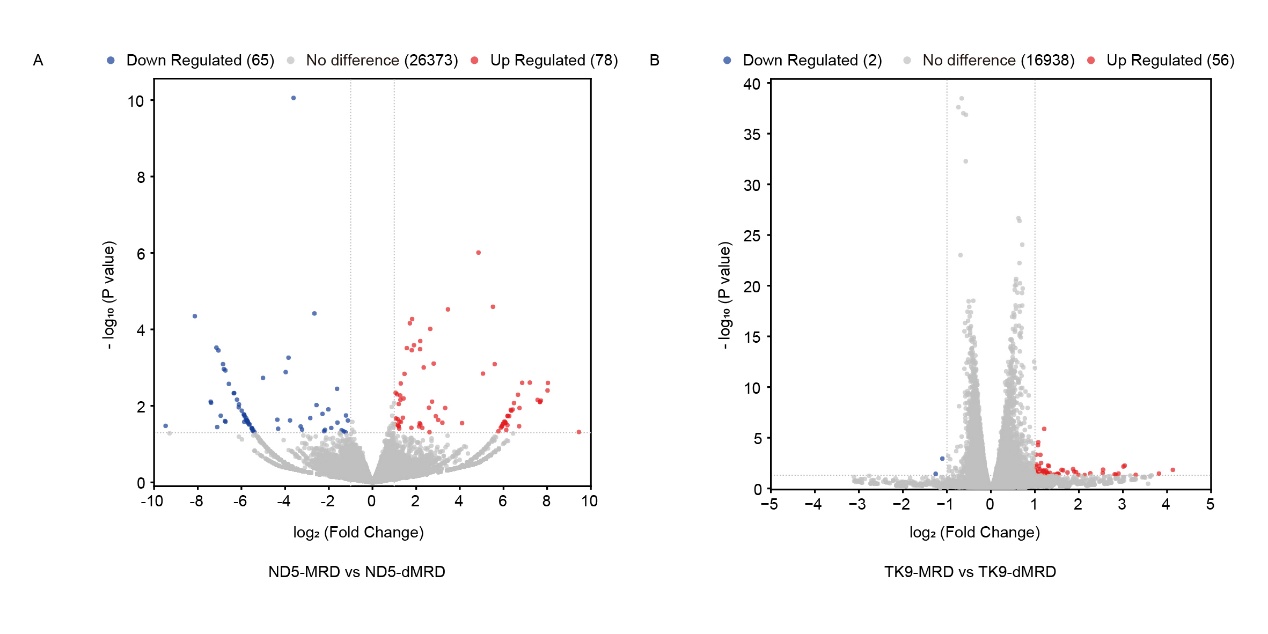


**Supplementary Figure S5. RNA-seq analysis after mitochondrial m^1^A editing by MRD.**

(A) Volcano plot displaying differentially expressed genes between ND5-MRD and ND5-dMRD samples. The x-axis represents log_2_ (fold change), and the y-axis shows –log_10_ (P value). Blue dots indicate significantly downregulated genes (65), red dots indicate significantly upregulated genes (78), and gray dots represent genes with no significant difference (26,373). The RNA-seq analysis was performed with two independent biological replicates.

(B) Volcano plot showing differentially expressed genes between TK9-MRD and TK9-dMRD samples. Axes and color scheme are consistent with (A). Blue dots represent significantly downregulated genes (2), red dots indicate significantly upregulated genes (56), and gray dots denote genes with no significant difference (16,938). The RNA-seq analysis was performed with two independent biological replicates.

**
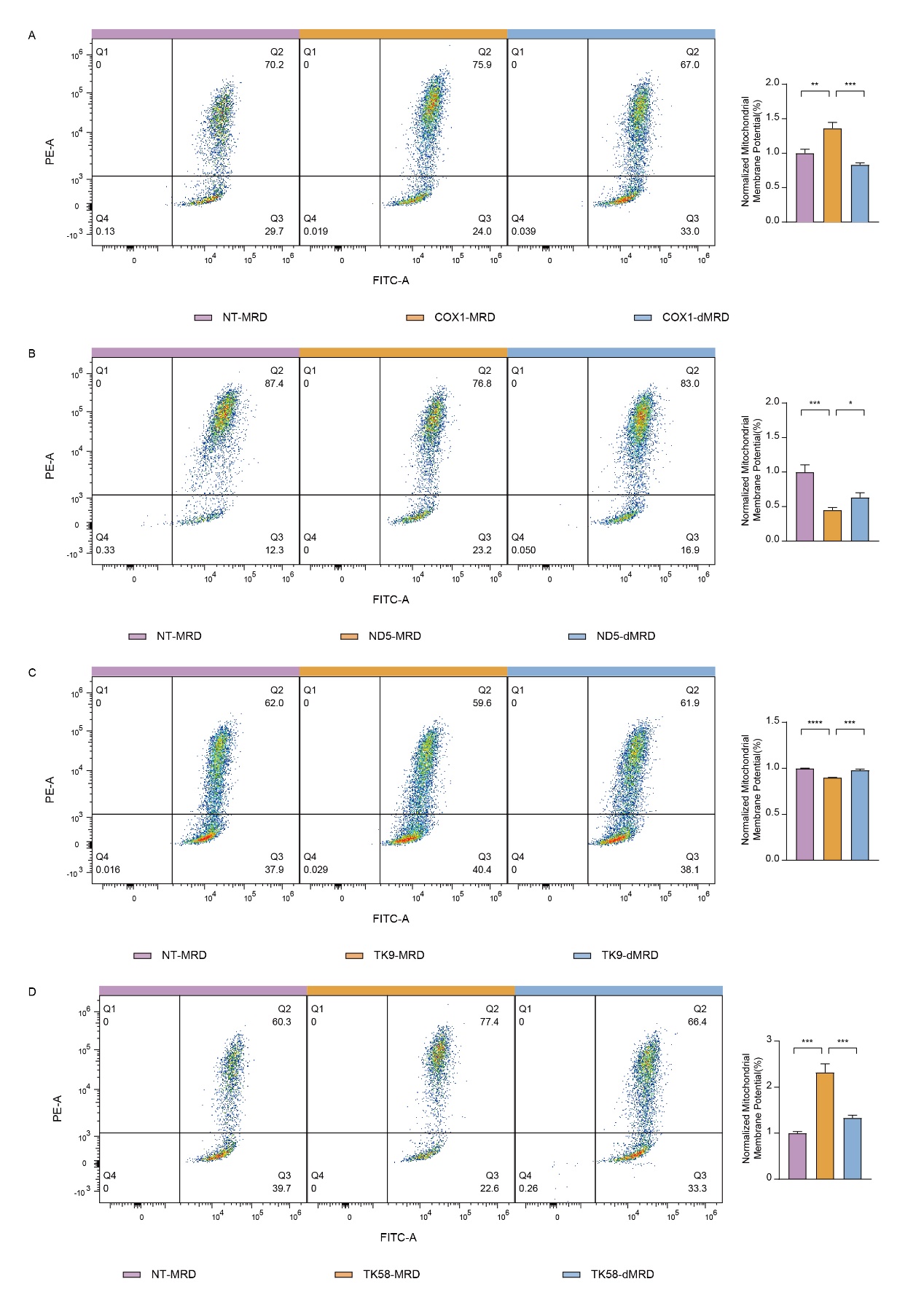
**

**Supplementary Figure S6. Mitochondrial membrane potential changes in MRD-edited HEK293T cells assessed by flow cytometry.**

(A-D) Flow cytometry analysis of mitochondrial membrane potential using a JC-1 assay in HEK293T cells edited with MRD constructs targeting different mitochondrial genes: (A) COX1, (B) ND5, (C)TK9, and (D)TK58. For each target, three conditions were compared: non-targeting control (NT-MRD, purple), MRD-edited (orange), and differentiated MRD-edited (dMRD, blue). The left panels show representative flow cytometry dot plots. The x-axis (FITC-A) represents the monomeric form of JC-1 (green fluorescence, indicating depolarized mitochondria), and the y-axis (PE-A) represents the J-aggregate form of JC-1 (red fluorescence, indicating polarized mitochondria). Quadrants are set to distinguish cells with high (Q2, red) and low (Q3, green) mitochondrial membrane potential. The percentage of cells in each quadrant is indicated. The right panels indicate the quantitative analysis of the normalized mitochondrial membrane potential (mean ± s.d. from three independent experiments). Data are presented as a percentage relative to the NT-MRD control set at 100%. Statistical significance was determined by the unpaired *t*-test (**p*< 0.05, ***p*< 0.01, ****p*< 0.001; ns, not significant).

**
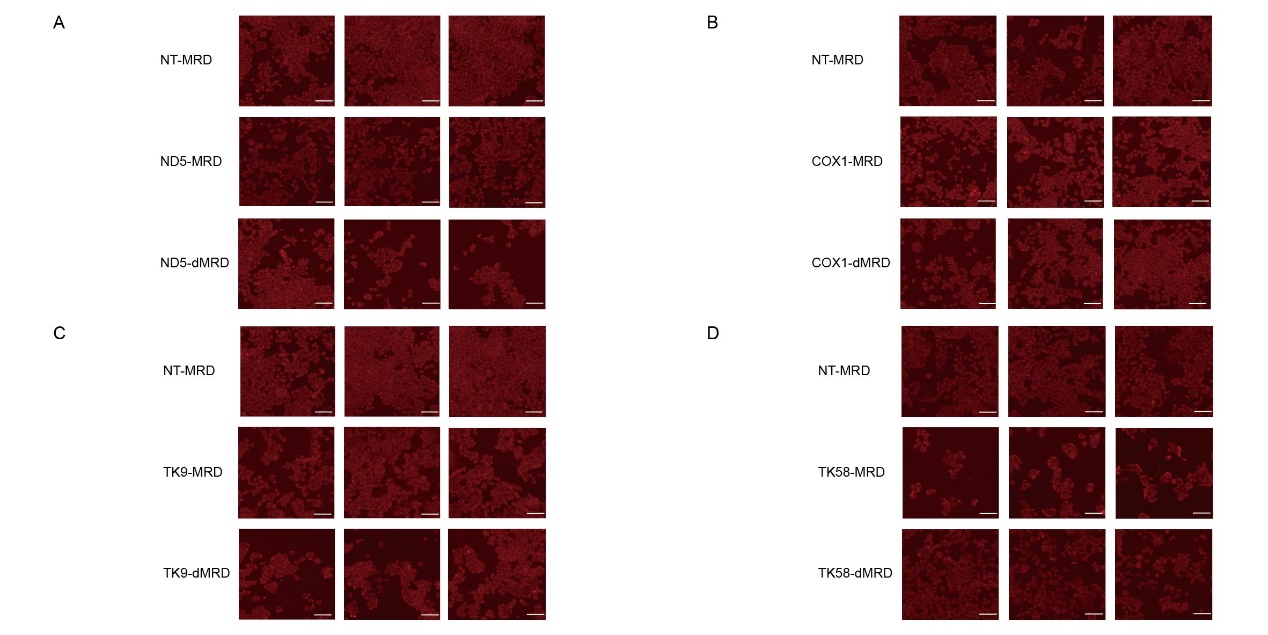
Supplementary Figure S7. Alteration of MMP by targeted m^1^A modification using the MRD editor.**

(A, B, C, D) MMP reflected by red signals, was visualized in HEK293T cells transfected with MRD targeted ND5 A13743 (A), COX1 A7375 (B), MT-TK9 (C), MT-TK58 (D) and control plasmids. Scale bar = 100 μm.


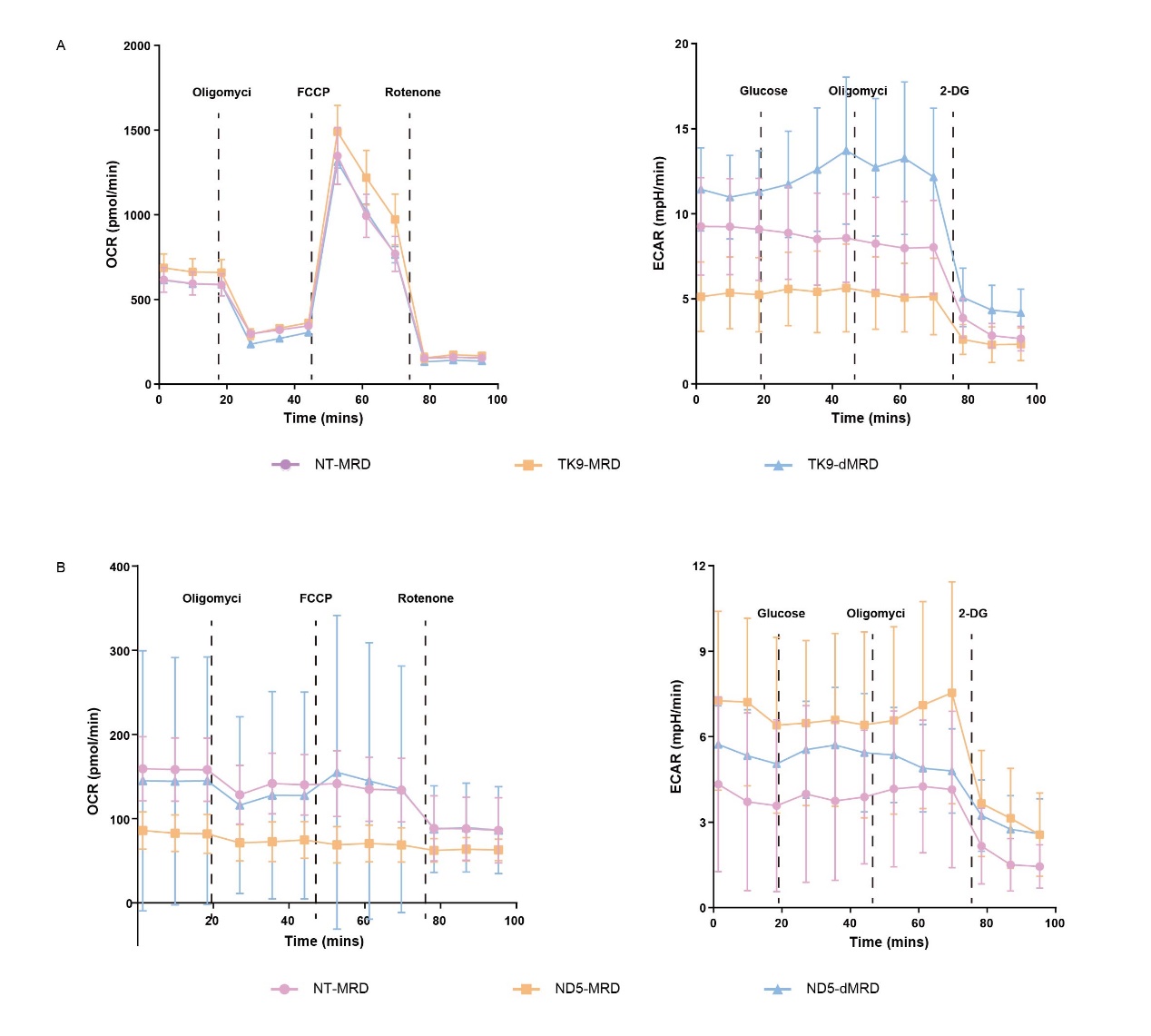


**Supplementary Figure S8. OCR and ECAR alterations in MRD-edited HEK293T cells quantified via Seahorse analysis.**

Oxygen consumption rate (OCR, left) and extracellular acidification rate (ECAR, right) measured in real-time by (A) TK9-MRD (orange) and TK9-dMRD (blue) cells compared to non-targeting control (NT-MRD, purple) and (B) ND5-MRD (orange) and ND5-dMRD (blue) cells compared to NT-MRD (purple). Data represent mean ± s.d. (n=3).


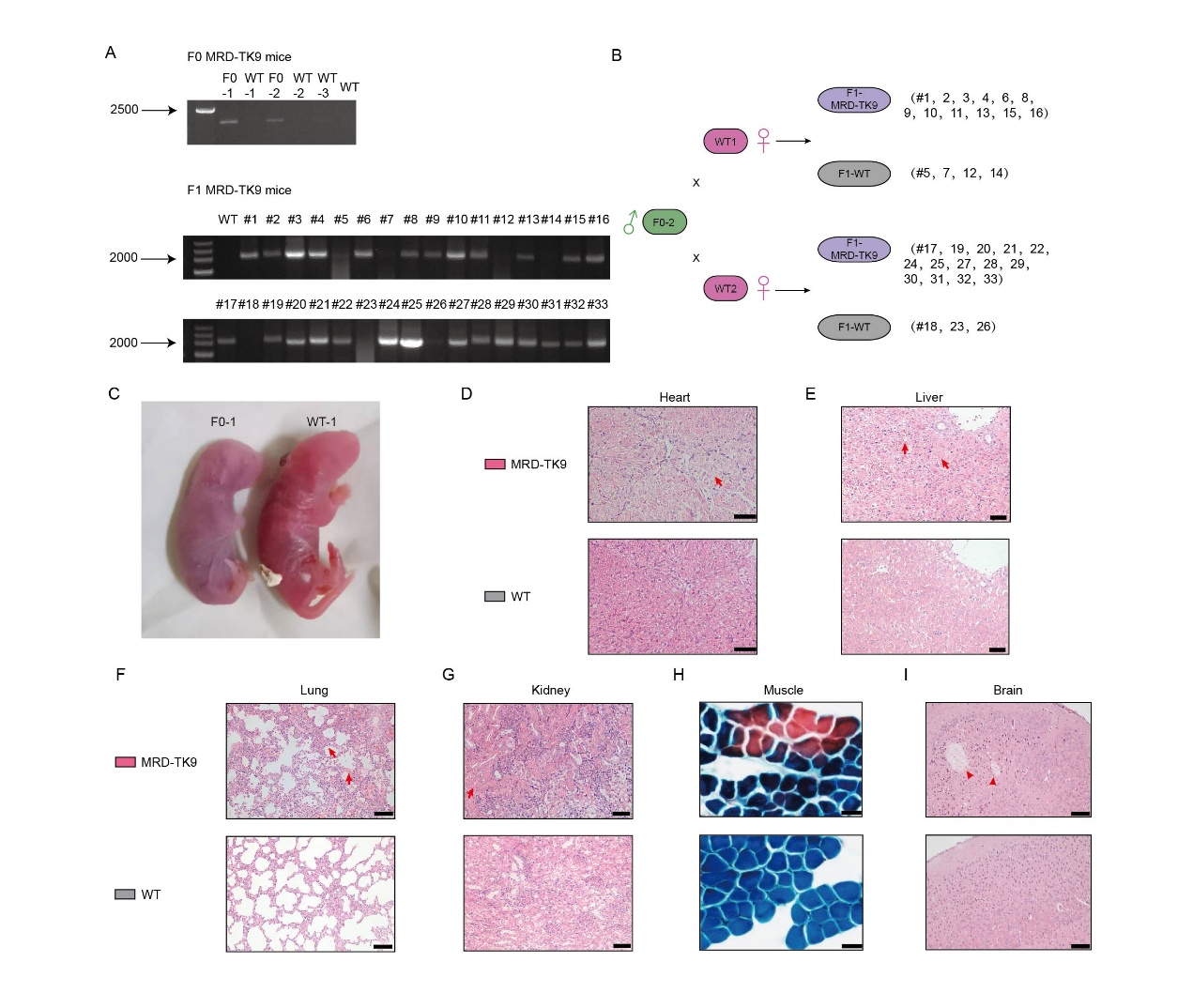


**Supplementary Figure S9. Demethylating m^1^A at mitochondrial tRNA-Lys A9 site in mice.**

(A) PCR identification of MRD sequence presence in F0 and F1 generation mice.

(B) Schematic illustrating the generation process of the F1 MRD-TK9 mice.

(C) Reduced body size and cyanosis of MRD-TK9 mice compared to WT mice, as shown by the image of the F0-1 (TK9-MRD) mouse (left) and WT-1 (WT) mouse (right).

(D) Significant histological changes in the heart, exhibiting significant cardiomyocyte hypertrophy in MRD-TK9 mice compared to WT mice. Scale bar = 50 μm.

(E) Significant histological changes in the liver, showing multiple small lipid droplets of variable size in the cytoplasm and hepatocellular necrosis in TK9-MRD mice compared to WT mice. Scale bar = 50 μm.

(F) Significant histological changes in the lung, demonstrating alveolar wall thickening in MRD-TK9 mice compared to WT mice. Scale bar = 100 μm.

(G) Significant histological changes in the kidney, revealing swollen tubular epithelial cells in MRD-TK9 mice compared to WT mice. Scale bar = 50 μm.

(H) Significant histological changes in the muscle, presenting ragged red fibers in TK9-MRD mice compared to WT mice by modified Gomori-trichrome staining. Scale bar = 25 μm.

(I) Significant histological changes in the brain, revealing the presence of pale areas in TK9-MRD mice compared to WT mice. Scale bar = 100 μm.


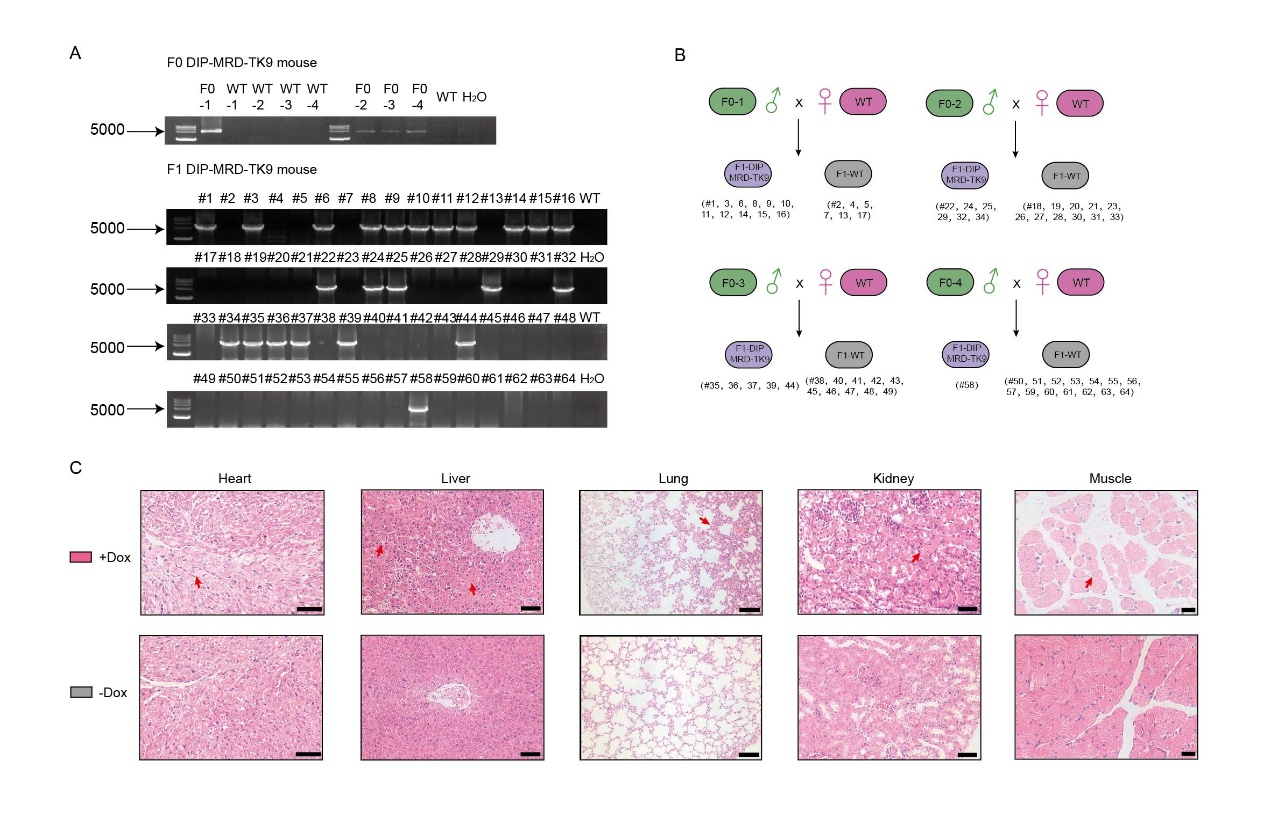


**Supplementary Figure S10. Dox-induced demethylating m^1^A at mitochondrial tRNA-Lys A9 site in mice.**

(A) PCR identification of MRD sequence presence in F0 and F1 generation mice.

(B) Schematic illustrating the generation process of the F1 TK9-DIP-MRD mice.

(C) Histological changes in multiple organs of Dox-induced TK9-DIP-MRD mice compared to WT mice. Scale bars = 50 μm (heart, liver, kidney, muscle) and 100 μm (lung).

**Supplementary Table 1. Primers used for plasmid construction in this study.**

| **PCR Primer** | **Sequence (5’-3’)** |
| --- | --- |
| PUF-R1-pcr-f | ggcaggagcaggcttttg |
| PUF-R1-pcr-r | aggcagcctggaggatttca |
| PUF-R2-pcr-f | tgaaatcctccaggctgcct |
| PUF-R2-pcr-r | gcctcgaatccgttctgcca |
| PUF-R3-pcr-f | tggcagaacggattcgaggc |
| PUF-R3-pcr-r | ggccatctagttcccgaacc |
| PUF-R4-pcr-f | ggttcgggaactagatggcc |
| PUF-R4-pcr-r | cctgtcccttaaacgcatcg |
| PUF-R5-pcr-f | cgatgcgtttaagggacagg |
| PUF-R5-pcr-r | gtgtgctggtgaagctcctc |
| PUF-R6-pcr-f | gaggagcttcaccagcacac |
| PUF-R6-pcr-r | tatcctcaggacgaccgtgc |
| PUF-R7-pcr-f | gcacggtcgtcctgaggata |
| PUF-R7-pcr-r | ccgtcgttcatggtgcacac |
| PUF-R8-pcr-f | gtgtgcaccatgaacgacgg |
| PUF-R8-pcr-r | ccctaagtcaacaccgttcttc |
| COX8-MTS-pcr-f | cagtgtggtggaattcatggcctccgttc |
| COX8-MTS-pcr-r | gcaccggttttcgcacgcggaactg |
| ALKBH3-pcr-f | ggaacggaggaaacgaattcatggaggaaaaaagacggcga |
| ALKBH3-pcr-r | gtttaaacgggccctctagagtcaccagggtgcccctc |
| ALKBH3-D193A-f | tggactggcacagtgctgatgaaccc |
| ALKBH3-D193A-r | gcactgtgccagtccacgctgtcctt |

**Supplementary Table 2. Primers used for RT-1306 method in this study.**

| **qPCR primer** | **Sequence (5’-3’)** |
| --- | --- |
| ND5-RT-primer | agttgaggtctagggctgtt |
| ND5-RT-1306-pcr-f | ggagtgagtacggtgtgcccccaccctactaaacccca |
| ND5-RT-1306-pcr-r | gagttggatgctggatggagttgaggtctagggctgtt |
| MT-TK-RT-primer | tcactgtaaagaggtg |
| MT-TK- RT-1306-pcr-f | ggagtgagtacggtgtgccactgtaa |
| MT-TK- RT-1306-pcr-r | gagttggatgctggatggtcactgtaaagaggtg |

**Supplementary Table 3. Primers used for SELECT method in this study.**

| **SELECT Primer** | **Sequence (5’-3’)** |
| --- | --- |
| ND5- m^1^A -select-f | tagccagtaccgtagtgcgtgtgcgaataggcttccggc |
| ND5- m^1^A -select-r | 5PHOS/gccaggcgtttaatggggcagaggctgagtcgctgcat |
| ND5- A -select-f | tagccagtaccgtagtgcgtgaaatcctgcgaataggct |
| ND5- A -select-r | 5PHOS/ccggctgccaggcgtttacagaggctgagtcgctgcat |
| COX1- m^1^A -select-f | tagccagtaccgtagtgcgtggggcatccatatagtcactccagg |
| COX1- m^1^A -select-r | 5PHOS/ttatggagggttcttctactattaggactcagaggctgagtcgctgcat |
| COX1- A -select-f | tagccagtaccgtagtgcgtggcatccatatagtcactccaggttta |
| COX1- A -select-r | 5PHOS/ggagggttcttctactattaggacttcagaggctgagtcgctgcat |
| MT-TK9- m^1^A -select-f | tagccagtaccgtagtgcgtgaaggttaatgctaagttagc |
| MT-TK9- m^1^A -select-r | 5PHOS/ttacagtgcagaggctgagtcgctgcat |
| MT-TK58- m^1^A -select-f | tagccagtaccgtagtgcgtgtggtcactgtaaagaggtgt |
| MT-TK58- m^1^A -select-r | 5PHOS/ggttctcttaatctttaactcagaggctgagtcgctgcat |
| MT-TK- A -select-f | tagccagtaccgtagtgcgtgagaggtgttggttctcttaa |
| MT-TK- A -select-r | 5PHOS/ctttaacttaaaaggttaatcagaggctgagtcgctgcat |
| mus-MT-TK9- m^1^A -select-f | tagccagtaccgtagtgcgtgaggttaacgctcttagc |
| mus-MT-TK9- m^1^A -select-r | 5PHOS/tcatagtgcagaggctgagtcgctgcat |
| mus-MT-TK9- A -select-f | tagccagtaccgtagtgcgtgctaactttaacttaaaaggt |
| mus-MT-TK9- A -select-r | 5PHOS/aacgctcttagcttcatagtcagaggctgagtcgctgcat |
| qpcr-f for select | atgcagcgactcagcctctg |
| qpcr-r for select | tagccagtaccgtagtgcgtg |

**Supplementary Table 4. Primers used for MRD-TK9 and DIP-MRD-TK9 mouse genotyping in this study.**

| **Genotyping primer** | **Sequence (5’-3’)** |
| --- | --- |
| Mus-MRD-TK9-f | cagtgtggtggaattcatggcctccgttc |
| Mus-MRD-TK9-r | gtttaaacgggccctctagagtcaccagggtgcccctc |
| Mus-DIP-MRD-TK9-f | gttgactgtgcctttaaacagcttg |
| Mus-DIP-MRD-TK9-r | gatctggccatctagagcggc |

**Supplementary Table 5. QPCR primers used in the study.**

| **qPCR primer** | **Sequence (5’-3’)** |
| --- | --- |
| ND5-qpcr-f | ctcatcgctacctccctgac |
| ND5-qpcr-r | agttgaggtctagggctgtt |
| COX1-qpcr-f | cgccgaccgttgactattct |
| COX1-qpcr-r | caggagtaggagagagggaggt |
| ACTB-qpcr-f | cgaggcccagagcaagaga |
| ACTB-qpcr-r | gtcatcttctcgcggttggc |

**Supplementary Note 1. Fused protein sequences of MRD**

MRD protein sequences used this study. Within MRD sequences, MTS sequences are highlighted in red, R1-R8 sequences of PUF are in black, ALKBH3 sequences are in purple.

>MTS-(ND5) PUF-ALKBH3 (8R-gcctattc)(+8):

MASVLTPLLLRGLTGSARRLPVPRAKGRSRLLEDFRNNRYPNLQLREIAGHIMEFSQDQHGSYFIRLKLERATPAERQLVFNEILQAAYQLMVDVFGNYVIQKFFEFGSLEQKLALAERIRGHVLSLALQMYGNYVIQKALEFIPSDQQNEMVRELDGHVLKCVKDQNGNHVVQKCIECVQPQSLQFIIDAFKGQVFALSTHPYGNYVIQRILEHCLPDQTLPILEELHQHTEQLVQDQYGSYVIRHVLEHGRPEDKSKIVAEIRGNVLVLSQHKFASYVVRKCVTHASRTERAVLIDEVCTMNDGPHSALYTMMKDQYASYVVEKMIDVAEPGQRKIVMHKIRPHIATLRKYTYGKHILAKLEKYYMKNGVDLGNGGNEFMEEKRRRARVQGAWAAPVKSQAIAQPATTAKSHLHQKPGQTWKNKEHHLSDREFVFKEPQQVVRRAPEPRVIDREGVYEISLSPTGVSRVCLYPGFVDVKEADWILEQLCQDVPWKQRTGIREDITYQQPRLTAWYGELPYTYSRITMEPNPHWHPVLRTLKNRIEENTGHTFNSLLCNLYRNEKDSVDWHSDDEPSLGRCPIIASLSFGATRTFEMRKKPPPEENGDYTYVERVKIPLDHGTLLIMEGATQADWQHRVPKEYHSREPRVNLTFRTVYPDPRGAPW*

>MTS-(ND5) PUF-ALKBH3 (8R-cctactaa)(-20):

MASVLTPLLLRGLTGSARRLPVPRAKGRSRLLEDFRNNRYPNLQLREIAGHIMEFSQDQHGSRFIQLKLERATPAERQLVFNEILQAAYQLMVDVFGCRVIQKFFEFGSLEQKLALAERIRGHVLSLALQMYGNYVIQKALEFIPSDQQNEMVRELDGHVLKCVKDQNGNHVVQKCIECVQPQSLQFIIDAFKGQVFALSTHPYGCRVIQRILEHCLPDQTLPILEELHQHTEQLVQDQYGNYVIQHVLEHGRPEDKSKIVAEIRGNVLVLSQHKFASYVVRKCVTHASRTERAVLIDEVCTMNDGPHSALYTMMKDQYASYVVRKMIDVAEPGQRKIVMHKIRPHIATLRKYTYGKHILAKLEKYYMKNGVDLGNGGNEFMEEKRRRARVQGAWAAPVKSQAIAQPATTAKSHLHQKPGQTWKNKEHHLSDREFVFKEPQQVVRRAPEPRVIDREGVYEISLSPTGVSRVCLYPGFVDVKEADWILEQLCQDVPWKQRTGIREDITYQQPRLTAWYGELPYTYSRITMEPNPHWHPVLRTLKNRIEENTGHTFNSLLCNLYRNEKDSVDWHSDDEPSLGRCPIIASLSFGATRTFEMRKKPPPEENGDYTYVERVKIPLDHGTLLIMEGATQADWQHRVPKEYHSREPRVNLTFRTVYPDPRGAPW*

>MTS-(ND5) PUF-ALKBH3 (8R-ggaagcct)(+4):

MASVLTPLLLRGLTGSARRLPVPRAKGRSRLLEDFRNNRYPNLQLREIAGHIMEFSQDQHGNYFIQLKLERATPAERQLVFNEILQAAYQLMVDVFGSYVIRKFFEFGSLEQKLALAERIRGHVLSLALQMYGSYVIRKALEFIPSDQQNEMVRELDGHVLKCVKDQNGSYVVEKCIECVQPQSLQFIIDAFKGQVFALSTHPYGCRVIQRILEHCLPDQTLPILEELHQHTEQLVQDQYGCRVIQHVLEHGRPEDKSKIVAEIRGNVLVLSQHKFASNVVEKCVTHASRTERAVLIDEVCTMNDGPHSALYTMMKDQYASYVVEKMIDVAEPGQRKIVMHKIRPHIATLRKYTYGKHILAKLEKYYMKNGVDLGNGGNEFMEEKRRRARVQGAWAAPVKSQAIAQPATTAKSHLHQKPGQTWKNKEHHLSDREFVFKEPQQVVRRAPEPRVIDREGVYEISLSPTGVSRVCLYPGFVDVKEADWILEQLCQDVPWKQRTGIREDITYQQPRLTAWYGELPYTYSRITMEPNPHWHPVLRTLKNRIEENTGHTFNSLLCNLYRNEKDSVDWHSDDEPSLGRCPIIASLSFGATRTFEMRKKPPPEENGDYTYVERVKIPLDHGTLLIMEGATQADWQHRVPKEYHSREPRVNLTFRTVYPDPRGAPW*

>MTS-(ND5) PUF-ALKBH3 (8R-gatttctc)(+20):

MASVLTPLLLRGLTGSARRLPVPRAKGRSRLLEDFRNNRYPNLQLREIAGHIMEFSQDQHGSYFIRLKLERATPAERQLVFNEILQAAYQLMVDVFGNYVIQKFFEFGSLEQKLALAERIRGHVLSLALQMYGSYVIRKALEFIPSDQQNEMVRELDGHVLKCVKDQNGNHVVQKCIECVQPQSLQFIIDAFKGQVFALSTHPYGNYVIQRILEHCLPDQTLPILEELHQHTEQLVQDQYGNYVIQHVLEHGRPEDKSKIVAEIRGNVLVLSQHKFACRVVQKCVTHASRTERAVLIDEVCTMNDGPHSALYTMMKDQYASYVVEKMIDVAEPGQRKIVMHKIRPHIATLRKYTYGKHILAKLEKYYMKNGVDLGNGGNEFMEEKRRRARVQGAWAAPVKSQAIAQPATTAKSHLHQKPGQTWKNKEHHLSDREFVFKEPQQVVRRAPEPRVIDREGVYEISLSPTGVSRVCLYPGFVDVKEADWILEQLCQDVPWKQRTGIREDITYQQPRLTAWYGELPYTYSRITMEPNPHWHPVLRTLKNRIEENTGHTFNSLLCNLYRNEKDSVDWHSDDEPSLGRCPIIASLSFGATRTFEMRKKPPPEENGDYTYVERVKIPLDHGTLLIMEGATQADWQHRVPKEYHSREPRVNLTFRTVYPDPRGAPW*

>MTS-(COX1) PUF-ALKBH3 (8R-gtgactat):

MASVLTPLLLRGLTGSARRLPVPRAKGRSRLLEDFRNNRYPNLQLREIAGHIMEFSQDQHGNYFIQLKLERATPAERQLVFNEILQAAYQLMVDVFGCRVIQKFFEFGSLEQKLALAERIRGHVLSLALQMYGNYVIQKALEFIPSDQQNEMVRELDGHVLKCVKDQNGNHVVQKCIECVQPQSLQFIIDAFKGQVFALSTHPYGCRVIQRILEHCLPDQTLPILEELHQHTEQLVQDQYGSYVIEHVLEHGRPEDKSKIVAEIRGNVLVLSQHKFANYVVQKCVTHASRTERAVLIDEVCTMNDGPHSALYTMMKDQYASYVVEKMIDVAEPGQRKIVMHKIRPHIATLRKYTYGKHILAKLEKYYMKNGVDLGNGGNEFMEEKRRRARVQGAWAAPVKSQAIAQPATTAKSHLHQKPGQTWKNKEHHLSDREFVFKEPQQVVRRAPEPRVIDREGVYEISLSPTGVSRVCLYPGFVDVKEADWILEQLCQDVPWKQRTGIREDITYQQPRLTAWYGELPYTYSRITMEPNPHWHPVLRTLKNRIEENTGHTFNSLLCNLYRNEKDSVDWHSDDEPSLGRCPIIASLSFGATRTFEMRKKPPPEENGDYTYVERVKIPLDHGTLLIMEGATQADWQHRVPKEYHSREPRVNLTFRTVYPDPRGAPW*

>MTS-(MT-TK9) PUF-ALKBH3 (8R-ttagcatt):

MASVLTPLLLRGLTGSARRLPVPRAKGRSRLLEDFRNNRYPNLQLREIAGHIMEFSQDQHGNYFIQLKLERATPAERQLVFNEILQAAYQLMVDVFGNYVIQKFFEFGSLEQKLALAERIRGHVLSLALQMYGCRVIQKALEFIPSDQQNEMVRELDGHVLKCVKDQNGNHVVQKCIECVQPQSLQFIIDAFKGQVFALSTHPYGSYVIERILEHCLPDQTLPILEELHQHTEQLVQDQYGCRVIQHVLEHGRPEDKSKIVAEIRGNVLVLSQHKFANYVVQKCVTHASRTERAVLIDEVCTMNDGPHSALYTMMKDQYANYVVQKMIDVAEPGQRKIVMHKIRPHIATLRKYTYGKHILAKLEKYYMKNGVDLGNGGNEFMEEKRRRARVQGAWAAPVKSQAIAQPATTAKSHLHQKPGQTWKNKEHHLSDREFVFKEPQQVVRRAPEPRVIDREGVYEISLSPTGVSRVCLYPGFVDVKEADWILEQLCQDVPWKQRTGIREDITYQQPRLTAWYGELPYTYSRITMEPNPHWHPVLRTLKNRIEENTGHTFNSLLCNLYRNEKDSVDWHSDDEPSLGRCPIIASLSFGATRTFEMRKKPPPEENGDYTYVERVKIPLDHGTLLIMEGATQADWQHRVPKEYHSREPRVNLTFRTVYPDPRGAPW*

>MTS-(MT-TK58) PUF-ALKBH3 (8R-ctttacag):

MASVLTPLLLRGLTGSARRLPVPRAKGRSRLLEDFRNNRYPNLQLREIAGHIMEFSQDQHGSYFIELKLERATPAERQLVFNEILQAAYQLMVDVFGCRVIQKFFEFGSLEQKLALAERIRGHVLSLALQMYGSYVIRKALEFIPSDQQNEMVRELDGHVLKCVKDQNGNHVVQKCIECVQPQSLQFIIDAFKGQVFALSTHPYGNYVIQRILEHCLPDQTLPILEELHQHTEQLVQDQYGNYVIQHVLEHGRPEDKSKIVAEIRGNVLVLSQHKFANYVVQKCVTHASRTERAVLIDEVCTMNDGPHSALYTMMKDQYASYVVRKMIDVAEPGQRKIVMHKIRPHIATLRKYTYGKHILAKLEKYYMKNGVDLGNGGNEFMEEKRRRARVQGAWAAPVKSQAIAQPATTAKSHLHQKPGQTWKNKEHHLSDREFVFKEPQQVVRRAPEPRVIDREGVYEISLSPTGVSRVCLYPGFVDVKEADWILEQLCQDVPWKQRTGIREDITYQQPRLTAWYGELPYTYSRITMEPNPHWHPVLRTLKNRIEENTGHTFNSLLCNLYRNEKDSVDWHSDDEPSLGRCPIIASLSFGATRTFEMRKKPPPEENGDYTYVERVKIPLDHGTLLIMEGATQADWQHRVPKEYHSREPRVNLTFRTVYPDPRGAPW*

>MTS-(mus-MT-TK9) PUF-ALKBH3 (8R-agcgttaa):

MASVLTPLLLRGLTGSARRLPVPRAKGRSRLLEDFRNNRYPNLQLREIAGHIMEFSQDQHGSRFIQLKLERATPAERQLVFNEILQAAYQLMVDVFGCRVIQKFFEFGSLEQKLALAERIRGHVLSLALQMYGNYVIQKALEFIPSDQQNEMVRELDGHVLKCVKDQNGNHVVQKCIECVQPQSLQFIIDAFKGQVFALSTHPYGSYVIERILEHCLPDQTLPILEELHQHTEQLVQDQYGSYVIRHVLEHGRPEDKSKIVAEIRGNVLVLSQHKFASNVVEKCVTHASRTERAVLIDEVCTMNDGPHSALYTMMKDQYACRVVQKMIDVAEPGQRKIVMHKIRPHIATLRKYTYGKHILAKLEKYYMKNGVDLGNGGNEFMEEKRRRARVQGAWAAPVKSQAIAQPATTAKSHLHQKPGQTWKNKEHHLSDREFVFKEPQQVVRRAPEPRVIDREGVYEISLSPTGVSRVCLYPGFVDVKEADWILEQLCQDVPWKQRTGIREDITYQQPRLTAWYGELPYTYSRITMEPNPHWHPVLRTLKNRIEENTGHTFNSLLCNLYRNEKDSVDWHSDDEPSLGRCPIIASLSFGATRTFEMRKKPPPEENGDYTYVERVKIPLDHGTLLIMEGATQADWQHRVPKEYHSREPRVNLTFRTVYPDPRGAPW*
